# Serum Arylesterase, Paraoxonase, and Lactonase Activities and Paraoxonase-1 Concentrations in Morbidly Obese Patients and Their Relationship with Non-Alcoholic Steatohepatitis

**DOI:** 10.3390/antiox12122038

**Published:** 2023-11-23

**Authors:** Helena Castañé, Andrea Jiménez-Franco, Cristian Martínez-Navidad, Cristina Placed-Gallego, Vicente Cambra-Cortés, Adelina-Miruna Perta, Marta París, Daniel del Castillo, Meritxell Arenas, Jordi Camps, Jorge Joven

**Affiliations:** 1 Unitat de Recerca Biomédica, Hospital Universitari de Sant Joan, Institut d’Investigació Sanitària Pere Virgili, Universitat Rovira i Virgili, Av. Dr. Josep Laporte 2, 43204 Reus, Spain; helena.castane@iispv.cat (H.C.); andrea.jimenez@urv.cat (A.J.-F.); cristian.martinez@iispv.cat (C.M.-N.); crisplaced@hotmail.com (C.P.-G.); vicente.cambra@urv.cat (V.C.-C.); adelina.miruna@iispv.cat (A.-M.P.); meritxell.arenas@urv.cat (M.A.); 2 Department of Bariatric Surgery, Hospital Universitari de Sant Joan, Institut d’Investigació Sanitària Pere Virgili, Universitat Rovira i Virgili, Av. Dr. Josep Laporte 2, 43204 Reus, Spain; marta.paris@salutsantjoan.cat (M.P.); danieldel.castillo@urv.cat (D.d.C.); 3 Department of Radiation Oncology, Hospital Universitari de Sant Joan, Institut d’Investigació Sanitària Pere Virgili, Universitat Rovira i Virgili, Av. Dr. Josep Laporte 2, 43204 Reus, Spain

**Keywords:** arylesterase, lactonase, morbid obesity, non-alcoholic fatty liver disease, non-alcoholic steatohepatitis, obesity, paraoxonase-1

## Abstract

Paraoxonase-1 (PON1) is an antioxidant enzyme associated with high-density lipoproteins (HDL). Reduced serum PON1 activity is found in diseases marked by oxidative stress and inflammation, but its role in obesity remains unclear. This study investigated PON1 activities and concentrations in morbidly obese individuals and explored the impacts of the genetic polymorphism PON1 rs662 and non-alcoholic fatty liver disease on enzymatic properties. We recruited 1349 morbidly obese patients undergoing bariatric surgery and 823 non-obese volunteers. PON1-related variables, including arylesterase, paraoxonase, and lactonase activities and PON1 concentrations, were examined. Our results showed that morbidly obese individuals exhibited higher PON1 concentrations but lower enzymatic activities than non-obese individuals. We observed inverse associations of arylesterase and paraoxonase activities with waist circumference (rho = −0.24, *p* < 0.001, and rho = −0.30, *p* < 0.001, respectively) and body mass index (rho = −0.15, *p* = 0.001, and rho = −0.23, *p* < 0.001), as well as direct associations of arylesterase, paraoxonase, and lactonase activities with HDL cholesterol (rho = 0.11, *p* = 0.005, rho = 0.20, *p* < 0.001, and rho = 0.20, *p* < 0.001). No significant differences were observed regarding metabolic syndrome, type 2 diabetes mellitus, hypertension, dyslipidemia, rs662 polymorphism allele frequencies, or the diagnosis of non-alcoholic steatohepatitis. Nevertheless, correlations were found between certain PON1-related variables, steatosis, and ballooning. In conclusion, changes in PON1-related variables in morbidly obese patients are dependent on the disease itself and HDL levels. The relationships between these variables and specific liver histological changes raise intriguing questions for consideration in future studies.

## 1. Introduction

One of the greatest epidemiological problems today is the rising prevalence of obesity, which has reached epidemic levels [1]. Obese individuals have a shared metabolic background, and their clinical condition is often affected by environmental influences, such as an imbalanced dietary pattern or a sedentary lifestyle [2]. Obesity is associated with chronic diseases, including type 2 diabetes mellitus, hepatic and cardiovascular diseases, and metabolic syndrome [3]. As these disorders are characterized by the overall presence of pro-inflammatory and pro-oxidant conditions [4], it is crucial to explore endogenous antioxidants and their potential roles as biomarkers or therapeutic targets [5].

Paraoxonase-1 (PON1) is an antioxidant enzyme that is widely distributed in human tissues and bound to high-density lipoproteins (HDL) in circulation [6]. Despite extensive research, a comprehensive understanding of the enzyme’s catalytic mechanisms is still out of reach. Presently, PON1 is regarded as a calcium-dependent ester hydrolase with remarkable versatility, capable of hydrolyzing a wide range of substrates, including esters, thioesters, phosphotriester carbonates, lactones, and thiolactones [6,7]. This versatility stems from three hydrolytic activities: (1) paraoxonase, which acts on the toxic organophosphate insecticide paraoxon; (2) arylesterase, targeting non-phosphorous aryl esters like phenyl acetate; and (3) lactonase, acting on lactones. These types of activity can be assessed in human serum using enzymatic assays with artificial substrates. Moreover, PON1 is subject to genetic polymorphisms, with the rs662 (Q192R) polymorphism being a strong determinant of enzymatic activity against some substrates. For example, the R allele is associated with greater catalytic activity against paraoxon in the general population [8,9].

Although the precise physiological substrate of PON1 is partly undefined, evidence suggests that PON1 exerts its antioxidant function by degrading specific oxidized cholesteryl esters and phospholipids, which result from oxidative stress. A model has been proposed to link this capability to PON1’s lactonase activity [10]. According to this model, oxidized lipids containing hydroxyl groups at the 5′-position may undergo lactonization by PON1, yielding lysophosphatidylcholine and δ-valerolactone products. Thus, based on this hypothesis, PON1’s ability to degrade lipid peroxides is secondary to its lipolactonase activity. In contrast, arylesterase and paraoxonase’s activities are directed toward synthetic chemicals, classifying them as promiscuous rather than as the enzyme’s primary functions [11]. 

Serum PON1 activity declines in non-communicable diseases characterized by oxidative stress and inflammation [6,12]. However, the available evidence regarding its involvement in the pathophysiology of obesity is limited and inconclusive. The enzyme’s promiscuous nature suggests that conclusions drawn from assays using a particular substrate may not be directly applicable to those obtained using other substrates. Another complicating factor is the fact that PON1 is primarily synthesized in the liver, and individuals with obesity often exhibit non-alcoholic fatty liver disease (NAFLD), which could affect the measurement results. This liver disease is a comorbidity often linked to obesity. NAFLD includes a broad spectrum of lesions, ranging from simple benign steatosis to more severe non-alcoholic steatohepatitis (NASH), which can then progress to fibrosis, cirrhosis, liver failure, and hepatocarcinoma [13], and it is currently the leading cause of liver transplantation [14]. Surprisingly, few studies have explored the influence of NAFLD on circulating PON1 levels [15,16,17,18].

Therefore, the primary aim of the current investigation was to characterize the alterations in serum PON1 concentrations and activity using various substrates within a large cohort of morbidly obese individuals. This study also clarified any plausible relationships between PON1-related variables and the occurrence of NAFLD. Furthermore, we elucidated the impact of the rs662 polymorphism on the enzymatic properties. We interpreted these results by applying advanced machine learning techniques, enabling us to gain deeper insights into the complex interactions and potential associations occurring within the data.

## 2. Materials and Methods

### 2.1. Study Design and Participants

We obtained a cross-sectional observational and comparative cohort that included 1349 consecutively recruited morbidly obese patients who were undergoing bariatric surgery in Hospital Universitari Sant Joan (Reus, Spain). All patients were over 18 years old and had a BMI > 35 kg/m^2^. They were also included in the Clinical Trials record NCT05554224. We obtained 12-h fasting blood samples immediately before surgery and an intraoperative liver wedge biopsy. The control group comprised 823 non-obese, healthy volunteers participating in an epidemiological study conducted in our geographical area. These volunteers were assessed within our hospital’s Preventive Medicine and Public Health Department, where a comprehensive blood analytical panel was completed. No clinical or laboratory indications of liver, kidney, neurological, psychiatric, thyroid, neoplastic, or infectious disease or chronic or acute inflammation were identified. Serum samples were stored at −80 °C in our institution’s biobank (Banc de Mostres Biològiques, Institut d’Investigació Sanitària Pere Virgili). 

### 2.2. Standard Laboratory Procedures

Serum glucose, insulin, total cholesterol, HDL cholesterol, and triglyceride concentrations, as well as aminotransferase activities, were determined via standard methods using a COBAS^®^ 8000 automated analyzer (Roche Diagnostics, Basel, Switzerland). Their intra- and inter-assay coefficients of variation were as follows: glucose, 0.7 and 1.2%; insulin, 2.0 and 2.8%; total cholesterol, 0.6 and 1.6%; HDL cholesterol, 0.9 and 1.8%; and triglycerides, 0.9 and 1.9%. Low-density lipoprotein (LDL) cholesterol and very-low-density lipoprotein (VLDL) cholesterol were calculated using the Friedewald formula [19]. The employed equations (data in mmol/L) were

VLDL cholesterol = Triglycerides/2.2

LDL cholesterol = Total cholesterol − VLDL cholesterol − HDL cholesterol

One hundred forty-four morbidly obese patients (10.7%) had serum triglyceride concentrations higher than 4.5 mmol/L (400 mg/dL), and their lipid profiles were excluded from statistical analyses since Friedewald’s formula is not valid for large hypertriglyceridemias [19]. A homeostatic model assessment of insulin resistance (HOMA-IR) was also devised [20].

Hepatic biopsy samples were embedded in paraffin and cut into 2 μm specimens for histological examination. Samples were then stained with Hematoxylin and Eosin, as well as Masson’s Trichrome. An expert hepatologist evaluated the slides for NASH diagnosis, following the criteria of Kleiner et al. [21]. Of all patients for whom histological data were available, we excluded 218 due to habitual alcohol intake and 300 because their tissue samples were insufficiently large for accurate evaluation to occur.

### 2.3. Measurement of PON1-Related Variables

Serum arylesterase (ARE) activity was assessed via the quantification of the enzymatic hydrolysis of phenylacetate at 270 nm and 25 °C. This process was conducted within a buffered solution containing 9 mM Tris–HCl (pH = 8.0) and supplemented with 0.9 mM CaCl_2_ [22]. Serum PON1 paraoxonase (PARX) activity was determined via the monitoring of the rate of paraoxon hydrolysis at 410 nm and 37 °C in a buffered solution comprising 0.05 mM glycine (pH = 10.5) supplemented with 1 mM CaCl_2_ [23]. Serum lactonase (LAC) activity was assessed by measuring the hydrolysis of 5-thiobutyl butyrolactone at a wavelength of 412 nm and 25 °C in a buffered solution containing 0.05 mM Tris–HCl (pH = 8.0) and supplemented with 1 mM CaCl_2_, as well as 0.5 mM 5,5′-dithio-bis-nitrobenzoic acid [24]. Chemical reagents were from Sigma Aldrich (Burlington, MA, USA). The serum PON1 concentration was determined using a commercially available ELISA (Elabscience Biotechnology Co., Ltd., Houston, TX, USA).

### 2.4. DNA Extraction and Genotyping 

For genotyping studies, whole blood was obtained from 796 controls and 800 patients and centrifuged at 1500× *g* and 4 °C. The buffy coat was incubated overnight with a cell lysis buffer. The ensuing protein was precipitated using a protein precipitation solution, separated via centrifugation, and subsequently discarded. We retained the resulting supernatant and treated it with cold isopropanol to induce DNA precipitation. After washing, the precipitated DNA was resuspended in nuclease-free water.

DNA was quantified using NanoDrop^TM^ equipment (Thermo Fisher Scientific, Waltham, MA, USA), ensuring DNA quality via the assessment of the 260 nm/280 nm ratio. We employed the Applied Biosystems TaqMan^TM^ SNP Genotyping Assays (Thermo Fisher Scientific) for PON1 rs662 genotyping. Next, 2 mg of DNA was dispensed into each well of a 384-well plate and dried overnight. Subsequently, the master mix and the requisite components from the kit were introduced into the wells. The probes, which specifically bound to the DNA helix’s minor groove, stabilized the probe–template complex, thus enhancing allelic discrimination.

### 2.5. Statistical Analyses and Machine Learning Data Interpretation

Statistical analyses were performed using RStudio (R version 4.0.2) and MetaboAnalyst 5.0. The programming codes used are shown in Table S1. The Shapiro–Wilk normality test was used to assess the normality of the PON1-related variables. Given the strong evidence disproving normality for these variables, we used non-parametric methods to ensure consistency (Table S2). We investigated the potential confounding effects of age and sex on the PON1-related variables. To assess the correlation between age and each PON1 variable, we used Spearman’s rank correlation coefficient. Similarly, to evaluate the differences in PON1 variables between sexes, we used the Mann–Whitney U test. The *p*-values obtained by performing these tests are depicted in Table S3. Upon identifying the significant associations of age and sex with certain types of PON1 activity, we adjusted our dataset to control for these confounding factors by fitting linear models for each variable. Two- and multi-group comparisons were performed using the Mann–Whitney and Kruskal–Wallis tests, respectively. For data management in R, the “Readxl” (version 1.4.3), “dplyr” (version 1.0.9), and “knitr” (version 1.39) packages were used. Significance and participants’ characteristics were assessed using the “Tableone” package (version 0.13.2), summarizing continuous variables as medians and interquartile ranges, while categorical variables were summarized as the number of individuals and percentages. For correlation analyses, we incorporated the “ggplot2” (version 3.3.6), “ggpur” (version 0.4.0), “corrplot” (version 0.92), and “Qgraph” (version 1.9.2) packages for univariate and multivariate correlations. For logistic regression, we utilized the “mice” package (version 3.14.0). Finally, MetaboAnalyst was used to obtain receiver operating characteristics (ROC) curves in two different modalities: classical ROC curves were obtained from the standard univariate mode, and a predictive model was developed using the tester mode. To implement the second mode, we split the dataset into training (75% of cases) and validation (25% of cases) sets. We combined the variables of interest using a linear supervised vector machine (SVM) algorithm, and the prediction test was repeated 100 times. 

## 3. Results

### 3.1. Clinical and Analytical Characteristics of Morbidly Obese Patients

Table 1 outlines the main clinical and analytical characteristics of the patients and members of the control group. We found significant differences in terms of age, sex distribution, the prevalence of type 2 diabetes mellitus, dyslipidemia, metabolic syndrome, and smoking and alcohol consumption habits. As predicted, there were notable distinctions with regard to BMI and waist circumference. Patients had higher levels of serum glucose, insulin, liver enzymes, and VLDL cholesterol and a higher HOMA-IR index. In contrast, they had lower levels of total cholesterol, HDL cholesterol, and LDL cholesterol than the members of the control group. In total, 194 morbidly obese patients were treated with statins.

### 3.2. Associations between PON1-Related Variables and the Metabolic Consequences of Obesity

**Figure 1:**
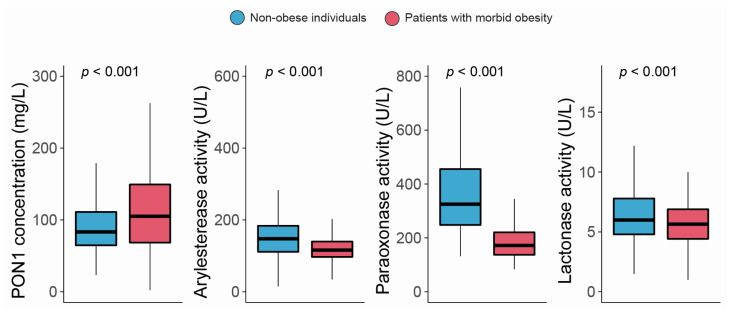
Paraoxonase-1-related variables in non-obese individuals and morbidly obese patients. PON1: paraoxonase-1.

**Figure 2:**
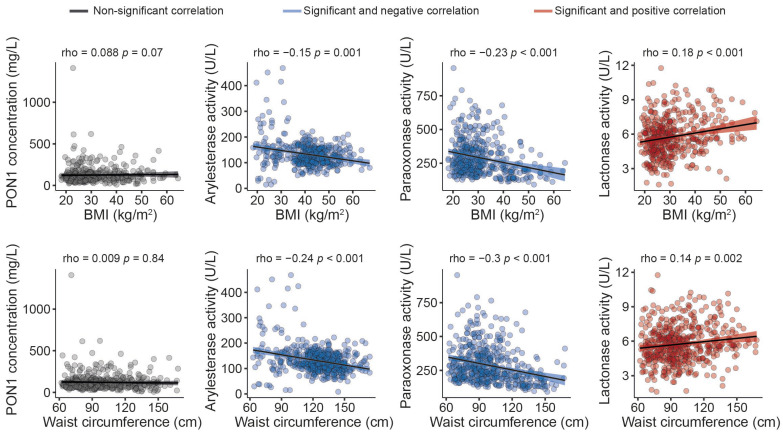
Relationships between paraoxonase-1-related variables and anthropometric measures. BMI: body mass index; PON1: paraoxonase-1.

**Figure 3:**
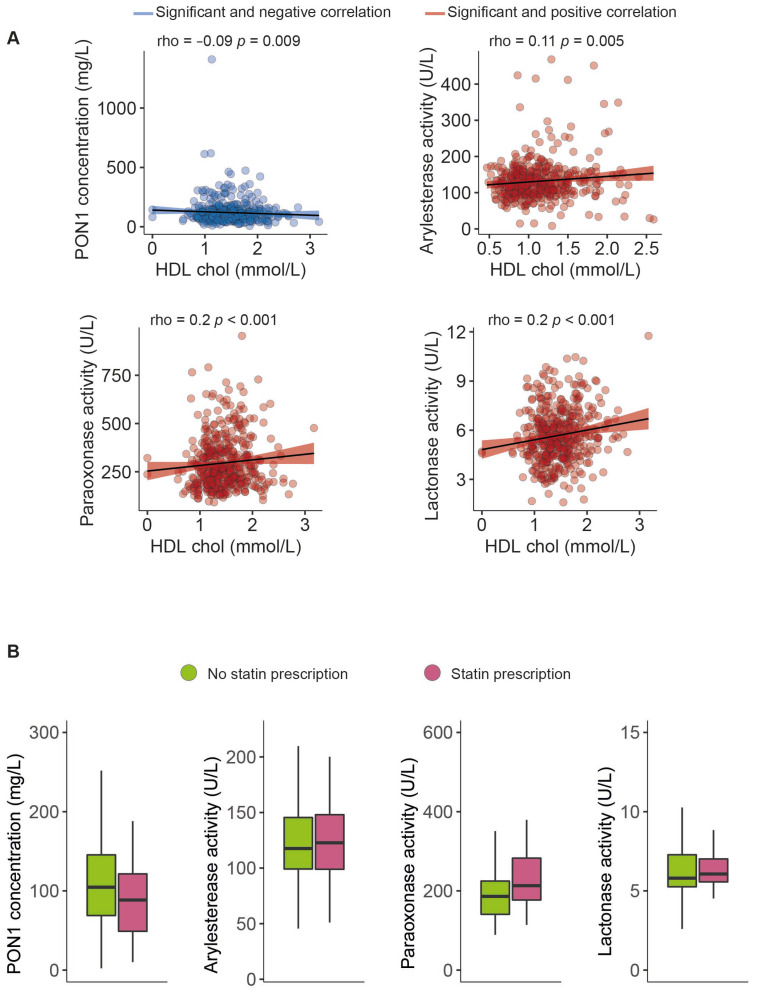
Paraoxonase-1-related variables related to high-density lipoprotein cholesterol concentrations (**A**) and statin prescriptions (**B**). HDL: high-density lipoprotein cholesterol; PON1: paraoxonase-1.

The serum PON1 concentration was significantly higher, and all the measured types of PON1 activity were markedly lower in morbidly obese patients than non-obese individuals (Figure 1). Moreover, we observed modest yet statistically significant inverse associations between ARE and PARX activities and waist circumference and BMI within the entire study population. In contrast, a positive correlation was established between the serum PON1 concentration, LAC activity, and BMI (Figure 2). We did not find any major differences in PON1-related variables in any groups due to metabolic syndrome, type 2 diabetes mellitus, arterial hypertension, or dyslipidemia (Table S4 and Figure S1). We also observed significant direct correlations between ARE, PARX, and LAC activities and HDL cholesterol concentrations (Figure 3A). PON1-related variables were not significantly influenced by statin treatment (Figure 3B). 

### 3.3. Influence of PON1 rs662 Genetic Polymorphism on PON1-Related Variables

**Figure 4:**
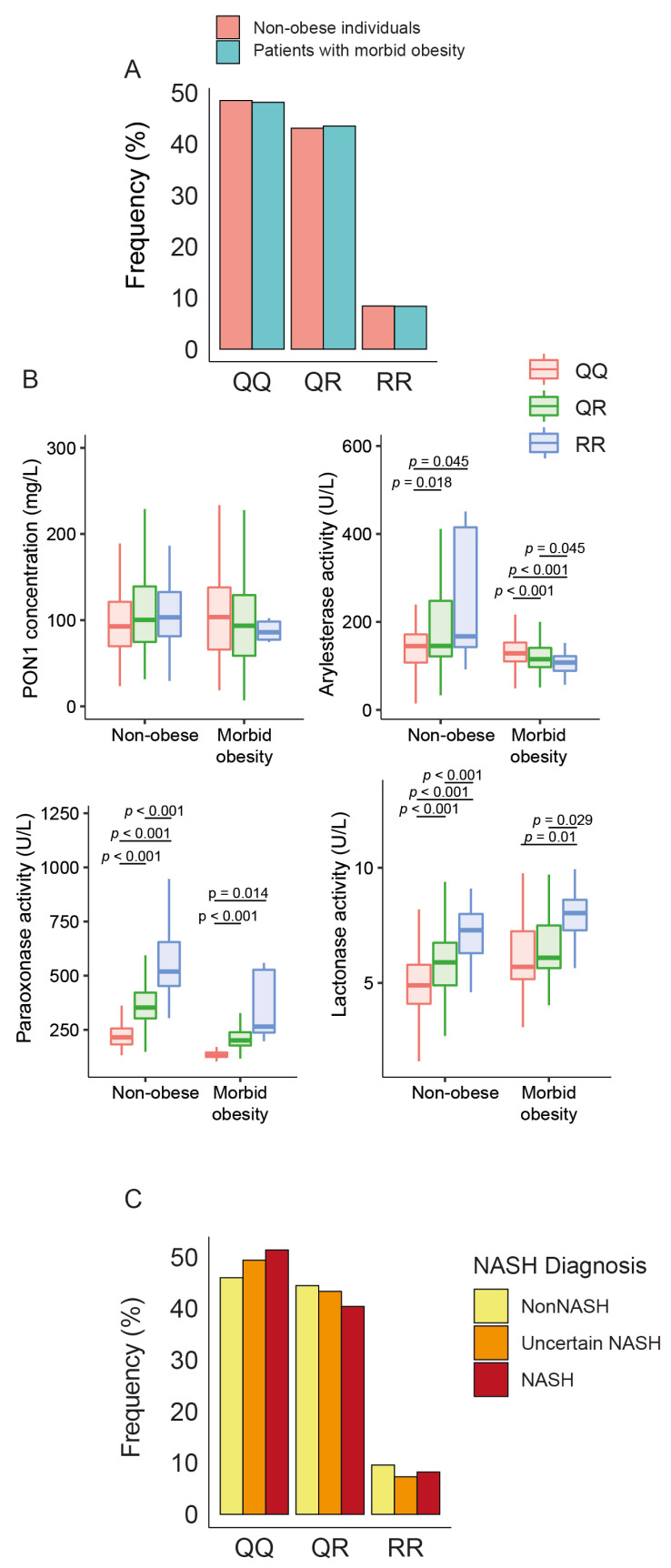
Impact of paraoxonase-1 (PON1) rs662 genetic polymorphism on PON1-related variables and liver status. (**A**) Frequency distribution of PON1 rs662 genotypes (QQ, QR, RR) in morbidly obese patients and non-obese participants. (**B**) Variation in PON1 concentrations and activity across the genotypes. (**C**) Frequency distribution of PON1 rs662 genotypes across patients with different liver statuses. NASH: non-alcoholic fatty liver disease.

There were no statistical differences in the frequency of PON1 rs662 polymorphism between patients and controls (Figure 4A). The allele R was associated with higher ARE, PARX, and LAC activities in non-obese individuals but only with PARX and LAC in obese patients. We did not observe any significant influence of this allele on serum PON1 concentrations (Figure 4B), nor did we find any significant differences in the frequency of rs662 polymorphism in patients with or without NASH (Figure 4C).

### 3.4. Relationships between PON1-Related Variables and the Degree of Liver Impairment

**Figure 5:**
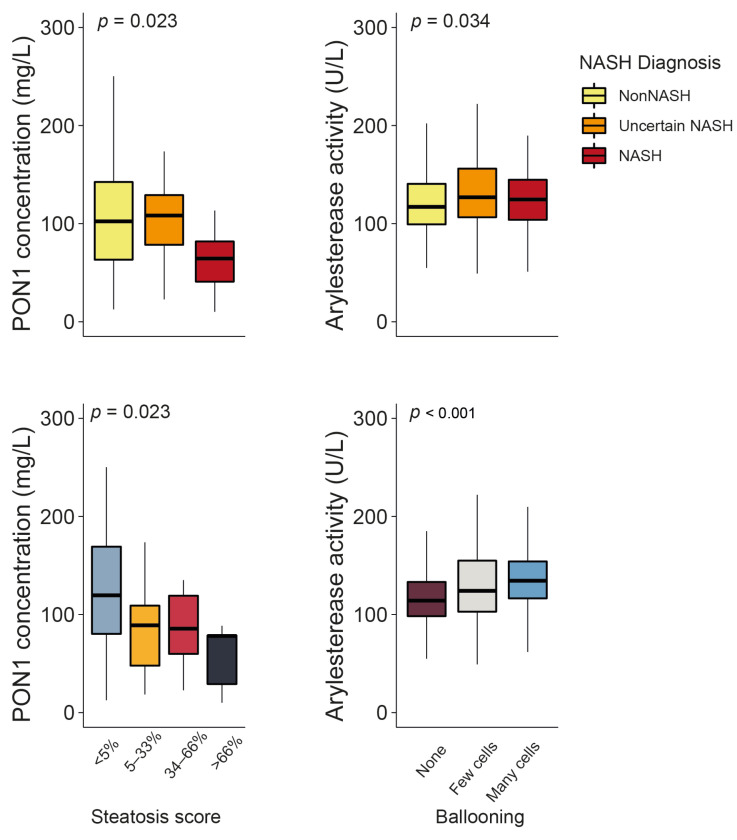
Paraoxonase-1 (PON1) concentration and arylesterase activity changed significantly depending on the degree of liver impairment. NASH: non-alcoholic steatohepatitis.

Serum PON1 concentrations were significantly lower in patients with obesity and NASH than in those without NASH (the clinical characteristics of these patients can be found in Table S5). They also decreased in tandem with the steatosis score. ARE activity showed a modest yet statistically significant increase in patients with uncertain NASH and NASH, as well as a more evident increase with regard to the number of ballooned hepatic cells (Figure 5). No other significant associations were identified between PON1-related parameters and the histological features of liver dysfunction (Figure S2), and none of these variables were usable as predictors of NASH (Figure S3).

## 4. Discussion

There is a discrepancy in the scientific literature regarding the changes in PON1-related variables associated with obesity. This discrepancy stems from inconclusive findings with regard to assessing various types of PON1 activity across diverse studies, limited sample sizes, variations in the severity of obesity under investigation, and failure to account for the potential impacts of genetic polymorphisms on PON1 activity [25,26].

One of the most complete studies is that of Cervellati et al. [27], who analyzed ARE, PARX, and LAC activities in 214 subjects with varying degrees of obesity, including 63 morbidly obese individuals. They compared these results with those of 101 overweight subjects and 129 individuals within the average weight range. They reported a reduction in ARE activity among morbidly obese patients but found no significant alterations in LAC or PARX activities. Additionally, there were no noteworthy differences in such activities in moderately obese individuals compared to those with a normal weight. Bacchetti et al. [28] identified reduced ARE, PARX, and LAC activities in 20 women with obesity, and Ferré et al. [26] identified diminished LAC while observing similar PARX in 110 children. Additionally, several studies by Bajnok et al. [29,30,31] reported low PARX activity in obese adults. However, other studies found no alterations in ARE, PARX, and LAC in patients with different degrees of obesity [32,33]. 

This study has focused on morbid obesity, a severe condition that significantly deteriorates the health and quality of life of affected individuals. We found lower ARE, PARX, and LAC activities compared to a cohort of non-obese individuals. This consistent reduction in the activity of these three distinct enzymatic functions of PON1 implies the shared suppression of the enzyme’s active site, irrespective of the substrate being processed. This suppression may be attributed to heightened oxidative stress, a trait associated with obesity [12]. Indeed, as we have previously mentioned [10], PON1 degrades lipoperoxides in lipoproteins and cells, although, to do so, the lipid molecule must form covalent bonds with the enzyme’s active site, leading to enzyme inactivation. The net result of oxidative stress will be low serum PON1 activity with high free radical levels, and the greater the oxidative stress, the lower the PON1 activity. This phenomenon, in turn, would explain the inconsistencies in PON1 activity alterations among overweight or moderately obese patients, presumably due to lower oxidative stress levels. These individuals tend to experience less conspicuous changes in PON1 activity, with some substrates potentially being more sensitive to stress than others, and influences from environmental factors may be more evident. This situation contrasts with the more pronounced alterations observed in individuals with extreme obesity.

Previous studies involving small groups of patients did not find any significant differences in PON1 concentrations between obese and non-obese subjects [34,35]. However, in this study, we identified a substantial increase in the serum concentration of this protein in morbidly obese patients. We view this increase as an attempt to counteract the decrease in activity. Our research group has observed this pattern in previous studies of patients with advanced liver disease [36,37] or infectious diseases [38]. We also reported that hepatic PON1 gene expression decreases in liver diseases. Nonetheless, this decrease occurs in the context of even greater protein degradation, resulting in enhanced PON1 protein expression [39].

We found weak but significant associations between PON1-related variables, waist circumference, BMI, and HDL cholesterol concentrations in the entire population. Nevertheless, modifications in PON1 were unaffected by the presence of hyperglycemia, dyslipidemia, or metabolic syndrome or the administration of statins. The interplay between obesity, type 2 diabetes mellitus, and metabolic syndrome is complex, and its influence on PON1 levels has not yet been defined. At present, most studies have consistently demonstrated reduced PON1 activity in individuals with type 2 diabetes mellitus and/or metabolic syndrome, regardless of the specific substrate used to perform the assessment [5,28,40,41,42,43]. Nevertheless, two investigations have reported comparable PON1 activity levels in specific subpopulations: one involved 40 Turkish, predominantly female, non-diabetic patients with both obesity and metabolic syndrome [33], and the other included 40 Taiwanese, non-diabetic men with obesity and metabolic syndrome [44], compared to their respective control groups. Our findings in morbidly obese patients align with the results previously reported by Cervellati et al. [27], who conducted a study involving a substantial patient cohort and evaluated PON1 activity using the same substrates used in our current investigation. Their results revealed a direct correlation between BMI, irrespective of age; hypertension; and type 2 diabetes mellitus status. Discrepancies in the outcomes of different studies may be associated with disparities in the demographic composition of the study populations, research objectives, sample sizes, and the analytical methodologies employed for PON1 assessment. Notably, in morbidly obese patients, the substantial impact induced via obesity and alterations in HDL levels may potentially eclipse the influence of comorbidities.

Exploring the relationship between PON1-related variables and the presence of liver alterations in patients with obesity is an area of great scientific interest. This is particularly true given the augmented risk of hepatic disease accompanying an elevated BMI [45]. In experimental investigations employing PON1-deficient murine models, exposure to a high-fat or high-cholesterol diet induced heightened oxidative stress and precipitated perturbations in metabolic processes, ultimately leading to the onset of hepatic steatosis [46].

Furthermore, several investigations have shown a significant reduction in PON1 activity among individuals afflicted by advanced liver disease compared to their healthy counterparts [23,36,37]. Nevertheless, this avenue of study still needs to be sufficiently explored, primarily due to the inherent challenges associated with procuring liver specimens for research. We took advantage of the fact that our participants were undergoing bariatric surgery for weight reduction, and, for a large number of these individuals, we had the opportunity to obtain intra-operative liver biopsies for analysis. 

We found that the serum PON1 concentration decreased in patients with NASH compared to those without NASH. This decrease depends on the degree of steatosis. The underlying cause of this association is beyond the scope of this study. However, it is consistent with a recent investigation [47] that obtained similar results by comparing 81 patients with NAFLD to 81 patients without NAFLD. These authors suggested that an imbalance in cytokine production could interfere with PON1 values but did not provide any explanation to justify this statement. They also admitted that it was impossible to identify whether a low concentration of PON1 is involved in the pathogenesis of NAFLD or one of the effects of NAFLD is the reduction in the concentration of PON1.

Regarding PON1 enzyme activity, we noticed a slight but significant increase in ARE levels in patients with NASH, which was associated with the number of ballooned hepatocytes. Ballooning is a hallmark of liver inflammation and related to increased endoplasmic reticulum stress, an unfolded protein response, and the Sonic Hedgehog pathway’s activation [48]. Indeed, we previously found a relationship between these metabolic pathways and hepatic expression and circulating levels of PON1 [49]. We accept that our observations regarding the relationships between PON1-related variables and the histopathological features of liver biopsies are limited in scope. Moreover, the differences were not substantial enough to identify PON1-related variables as biomarkers of NASH in morbid obesity. However, we believe that these findings serve as a strong motivation for additional investigation and may contribute to clarifying the potential role of PON1 in NAFLD.

Previous studies examined PON1 levels in NASH patients compared to healthy individuals, with some reporting lower levels [50,51], similar levels [52], or higher levels [53] of ARE or PARX activity. Recently, van den Berg et al. [16] conducted a comprehensive investigation of the impact of NAFLD on serum ARE levels, which involved over 7000 participants in a population-based study. Their findings indicated that the enzymatic activity of ARE remained unaltered in the presence of NAFLD. However, it is noteworthy that the diagnosis of liver disease in their study was established not through histological biopsy examination but via the assessment of the fatty liver index, a computed value based on various serum parameters, waist circumference, and BMI. This disparity between each methodology and our observation that the fluctuations that we identified in ARE activity were minimal could account for the varying conclusions reached by each investigation.

The rs662 polymorphism in the coding region of the PON1 gene is a pivotal determinant of enzymatic activity in serum. Individuals homozygous for the R allele (carrying Gln at position 192) have PON1 activity that is several magnitudes higher than that of Q homozygotes (Arg at position 192), while heterozygotes have intermediate values [54,55,56]. In contrast, this polymorphic variation exerts a minimal influence on the concentration of PON1 within the serum, underscoring the divergence between the enzyme concentration and its catalytic activity [57]. The importance of the PON1 genotypes was evidenced when some studies reported an association between R and L alleles and a higher risk of cardiovascular disease [6]. We did not find any differences in the frequencies of the different isoforms of the rs662 polymorphism between patients and the control group or regarding the degree of liver alteration. This finding indicates that differences in allele frequencies between morbidly obese patients and controls do not explain the differences observed in the levels of enzymatic activity. These isoforms generally influenced enzymatic activity in the expected manner, with the R allele being associated with higher activity. However, it is interesting to note that this association was lost for ARE in obese patients, an unprecedented observation for which, at present, we can find no explanation.

Our study prompts the question of which substrate is most suitable for the assessment of circulating PON1 activity. While the potential variance in enzymatic activity across diverse substrates in the context of different diseases remains uncertain, our findings in morbid obesity show consistent outcomes between various substrates. From our perspective, if PON1’s native activity is that of a lactonase, as indicated by some studies [10,11], with esterase activity being an acquired or promiscuous trait, lactones emerge as optimal substrates for pathophysiological investigations. In contrast, for toxicological inquiries, the use of paraoxon or phenylacetate may assume greater significance. Our results and those of other researchers underscore the importance of substrate selection, which should be judiciously considered depending on the specific research objectives.

This study has several limitations. Firstly, it was a single-center investigation conducted within a Caucasian population in Southwestern Europe. Several prior studies have shown that PON1 activity in serum is dependent on ethnicity [58,59,60,61]. Furthermore, the potential impacts of environmental and dietary factors cannot be discounted. Consequently, our study’s findings may not be readily extrapolated to the global population. 

Secondly, this study is a cross-sectional observational study, which, while effective at establishing the status of circulating PON1 levels in morbidly obese individuals in our specific population, leads to unaddressed queries due to its inherent design constraints. The lack of influence of the rs662 polymorphism on ARE levels among our patients and the relationships between PON1-related parameters, hepatic steatosis, and hepatic ballooning are noteworthy observations that presently lack comprehensive explanations and warrant further scrutiny. 

In addition, our results are restricted to extremely obese patients. We believe that this is a different disease to light or moderate obesity, thus having other metabolic implications. The size of the adipocytes in these patients is enormous, and lipid metabolism is hugely altered [62,63], which undoubtedly affects oxidative stress levels and lipoprotein synthesis. Therefore, alterations in the levels of PON1-related variables may not be the same as those occurring in milder obesity. However, this limitation highlights the relevance of our results, as understanding the complex changes in metabolic dysfunction in extreme obesity is a challenging but essential step in improving the health outcomes of affected individuals.

## 5. Conclusions

This study demonstrates that morbidly obese patients experience a consistent decrease in serum PON1 activity, as measured via three different assays and an increase in PON1 concentration. These alterations depend on severe obesity itself and HDL levels, and they do not depend on the degree of liver impairment, comorbidities, or the frequencies of the different isoforms of the rs662 polymorphism. Moreover, some results, such as the intriguing inverse relationship between the serum PON1 concentration and the degree of steatosis or the loss of influence of the R allele in ARE activity in patients with obesity, raise new questions and merit further scientific investigation.

## Figures and Tables

**Table 1:** Clinical and analytical characteristics of non-obese individuals and morbidly obese patients.

	Non-Obese Patients (n = 823)	Morbidly Obese Patients (n = 1349)	*p*-Value
Sex, female, n (%)	431 (52.9)	915 (73.1)	<0.001
Age (years)	43.0 (32.0–56.0)	49.0 (41.0–57.0)	<0.001
BMI (kg/m^2^)	26.48 (23.24–29.96)	44.38 (40.65–48.95)	<0.001
Waist circumference (cm)	90 (79–99)	131 (121–140)	<0.001
Smoking, n (%)			<0.001
Non-smokers	543 (66.0)	1107 (82.1)	
Smokers	280 (34.0)	242 (17.9)	
Drinking, n (%)			0.001
Non-habitual drinkers	647 (78.6)	1131 (83.8)	
Drinkers	176 (21.4)	218 (16.2)	
T2DM, n (%)	41 (5.0)	307 (22.8)	<0.001
HT, n (%)	107 (13.0)	513 (38.0)	<0.001
DLP, n (%)	58 (7.0)	289 (21.4)	<0.001
Metabolic syndrome, n (%)	111 (13.5)	482 (35.7)	<0.001
Hyperthyroidism, n (%)	0	105 (7.8)	-
Hypothyroidism, n (%)	0	5 (0.4)	-
Cancer, n (%)	0	0	-
Statin prescription, n (%)	0 (0.0)	194 (14.4)	<0.001
Glucose (mmol/L)	4.70 (4.30–5.20)	6.72 (5.50–8.49)	<0.001
Insulin (pmol/L)	49.42 (31.93–70.05)	71.77 (39.59–123.45)	<0.001
HOMA-IR	1.48 (0.95–2.31)	3.33 (1.72–6.08)	<0.001
Triglycerides (mmol/L)	1.10 (0.70–1.60)	1.48 (1.15–2.04)	<0.001
Total cholesterol (mmol/L)	5.20 (4.60–5.90)	4.14 (3.57–4.80)	<0.001
HDL cholesterol (mmol/L)	1.46 (1.23–1.74)	1.01 (0.84–1.27)	<0.001
LDL cholesterol (mmol/L)	3.12 (2.53–3.76)	2.53 (1.97–3.13)	<0.001
VLDL cholesterol (mmol/L)	0.50 (0.32–0.73)	0.67 (0.52–0.93)	<0.001
ALT (μKat/L)	0.32 (0.23–0.44)	0.56 (0.39–0.88)	<0.001
AST (μKat/L)	0.34 (0.28–0.43)	0.56 (0.39–0.83)	<0.001
GGT (μKat/L)	0.24 (0.16–0.39)	0.35 (0.23–0.56)	<0.001

ALT: alanine aminotransferase; AST: aspartate aminotransferase; BMI: body mass index; DLP: dyslipidemia; GGT: gamma-glutamyl transferase; HDL: high-density lipoprotein; HOMA-IR: homeostasis model assessment for insulin resistance; HT: hypertension; LDL: low-density lipoprotein; T2DM: type 2 diabetes mellitus; VLDL: very-low-density lipoprotein.

## Data Availability

All data presented in this study are available upon request.

## Supplementary Materials

The following supporting information can be downloaded at: https://www.mdpi.com/article/10.3390/antiox12122038/s1, Table S1: Programming codes in R; Table S2: *p*-values from the Shapiro–Wilk normality test for PON1-related variables; Table S3: Age and sex were tested as confounding factors for PON1-related variables; Table S4: Linear regression analysis showing the relationship between PON1 variables and metabolic syndrome, type 2 diabetes mellitus, hypertension, and dyslipidemia as a set; Figure S1: Paraoxonase-1 (PON1)-related variables in non-obese individuals and morbidly obese patients segregated according to comorbidities; Table S5: Clinical, analytical, and histologic characteristics of morbidly obese patients and different degrees of liver severity; Figure S2: Paraoxonase-1 (PON1)-related variables in morbidly obese patients segregated according to the histopathological characteristics of liver biopsies; Figure S3: Paraoxonase-1 (PON1) concentration and activity were not able to predict NASH in morbidly obese patients. (A) Receiver operating characteristics (ROC) of PON1-related variables. (B) ROC from the predictive model built with linear supervised vector machine (SVM) and its accuracy.
