# Supplementary material for: Serum Arylesterase, Paraoxonase, and Lactonase Activities and Paraoxonase-1 Concentrations in Morbidly Obese Patients and Their Relationship with Non-Alcoholic Steatohepatitis
Source: Antioxidants (Basel). 2023 Nov 23;12(12):2038. doi: 10.3390/antiox12122038 (PMC10741051; doi:10.3390/antiox12122038)
Supplement: Supplementary file 1 [file antioxidants-12-02038-s001.zip › antioxidants-2694667-supplementary.pdf]

Table S1. Programming codes in R.

Characteristics of the population

```
vars <- c("Sex", "Age", "BMI", "Heart_rate", "TAS", "TAD", "Pcadera",
"Pcintura", "Smoking", "Drinking", "T2DM", "HT", "DLP", "Metabolic_syn
", "Statins", "Glucose", "Insulin", "HOMAIR", "TG", "CHOL", "HDL", "LD
L", "VLDL", "ALT", "AST", "GGT")
catvars <- c("Sex", "Smoking", "Drinking", "T2DM", "HT", "DLP", "Metab
olic_syn", "Statins")
table_1 <- CreateTableOne(data = Db, vars = vars, factorVars = catvars
, strata = "Group")
print(table_1, nonnormal = vars, formatOptions = list(big.mark = ","))
```

Normality test for PON1 variables

```
p_values <- sapply(pon1vars, function(var) {
  shapiro.test(Db[[var]])$p.value
})

suppl_table_1 <- data.frame(P_Value = p_values)

print(suppl_table_1)
```

Confounding factors

Correlation between age and PON1

```
test_correlation <- function(variable_name, data) {
  correlation <- cor.test(data$Age, data[[variable_name]], method="spe
arman")
  return(data.frame(variable = variable_name, p_value = correlation$p.
value))
}

suppl_table_2a <- do.call(rbind, lapply(pon1vars, test_correlation, da
ta = Db))

print(suppl_table_2a)
```

Correlation between sex and PON1

```
test_sex_difference <- function(variable_name, data) {
  test <- wilcox.test(data[[variable_name]] ~ data$Sex)
  return(data.frame(variable = variable_name, p_value = test$p.value))
}

suppl_table_2b <- do.call(rbind, lapply(pon1vars, test_sex_difference,
data = Db))

print(suppl_table_2b)
```

Adjusting the dataset

```
lm_Paraxonase <- lm(Paraxonase ~ Sex, data = Db, na.action = "na.exclu
de")
Db$adj_Paraxonase <- residuals(lm_Paraxonase) + mean(na.omit(Db$Paraxo
nase), na.rm = TRUE)

lm_Lactonase <- lm(Lactonase ~ Age, data = Db, na.action = "na.exclude
")
Db$adj_Lactonase <- residuals(lm_Lactonase) + mean(na.omit(Db$Lactonas
e), na.rm = TRUE)

adjpon1vars <- c("PON1c", "ARE_act_2022", "adj_Paraxonase", "adj_Lacto
nase")
```

PON1 and obesity

```
plot <- ggplot(Db, aes(x = Group, y = PON1c, fill = Group)) +
  geom_boxplot(outlier.shape=NA, color = "black", size = 0.5) +
  stat_compare_means(label = "p", tip.length = 0, size = 4) +
  scale_fill_manual(values = c("#41a8d1", "#e3596d"),
                    labels = c("Non-obese", "Patients with morbid obes
ity")) +
  ylim(0, 300) +
  xlab("") +
  ylab("PON1 concentration (mg/L)") +
  theme_classic() +
  theme(axis.line = element_line(colour = "black", size = 0.5),
        panel.background = element_rect(fill = "white"),
        panel.grid.major = element_blank(),
        panel.grid.minor = element_blank(),
        axis.text = element_text(size = 12),
        axis.text.x = element_blank(),
        axis.ticks.x = element_blank(),
        axis.title = element_text(size = 14, face = "bold"),
        legend.position =  "none",
        axis.ticks = element_line(colour = "black", size = 0.5),
        aspect.ratio = 2)
ggsave(filename = "Raw plots/Obesity/PON1c.pdf",
       plot = plot,
       device = "pdf",
       height = 3,
       width = 3)

plot <- ggplot(Db, aes(x = Group, y = ARE_act_2022, fill = Group)) +
  geom_boxplot(outlier.shape=NA, color = "black", size = 0.5) +
  stat_compare_means(label = "p", tip.length = 0, size = 4) +
  scale_fill_manual(values = c("#41a8d1", "#e3596d"),
                    labels = c("Non-obese", "Patients with morbid obes
ity")) +
  ylim(0, 600) +
  xlab("") +
  ylab("Arylesterease activity (U/L)") +
  theme_classic() +
  theme(axis.line = element_line(colour = "black", size = 0.5),
```

```
        panel.background = element_rect(fill = "white"),
        panel.grid.major = element_blank(),
        panel.grid.minor = element_blank(),
        axis.text = element_text(size = 12),
        axis.text.x = element_blank(),
        axis.ticks.x = element_blank(),
        axis.title = element_text(size = 14, face = "bold"),
        legend.position =  "none",
        axis.ticks = element_line(colour = "black", size = 0.5),
        aspect.ratio = 2)
ggsave(filename = "Raw plots/Obesity/AREact.pdf",
       plot = plot,
       device = "pdf",
       height = 3,
       width = 3)

plot <- ggplot(Db, aes(x = Group, y = adj_Paraxonase, fill = Group)) +
  geom_boxplot(outlier.shape=NA, color = "black", size = 0.5) +
  stat_compare_means(label = "p", tip.length = 0, size = 4) +
  scale_fill_manual(values = c("#41a8d1", "#e3596d"),
                    labels = c("Non-obese", "Patients with morbid obes
ity")) +
  ylim(0, 800) +
  xlab("") +
  ylab("Paraoxonase activity (U/L)") +
  theme_classic() +
  theme(axis.line = element_line(colour = "black", size = 0.5),
        panel.background = element_rect(fill = "white"),
        panel.grid.major = element_blank(),
        panel.grid.minor = element_blank(),
        axis.text = element_text(size = 12),
        axis.text.x = element_blank(),
        axis.ticks.x = element_blank(),
        axis.title = element_text(size = 14, face = "bold"),
        legend.position =  "none",
        axis.ticks = element_line(colour = "black", size = 0.5),
        aspect.ratio = 2)
ggsave(filename = "Raw plots/Obesity/Paraxact.pdf",
       plot = plot,
       device = "pdf",
       height = 3,
       width = 3)

plot <- ggplot(Db, aes(x = Group, y = adj_Lactonase, fill = Group)) +
  geom_boxplot(outlier.shape=NA, color = "black", size = 0.5) +
  stat_compare_means(label = "p", tip.length = 0, size = 4) +
  scale_fill_manual(values = c("#41a8d1", "#e3596d"),
                    labels = c("Non-obese", "Patients with morbid obes
ity")) +
  ylim(0, 18) +
  xlab("") +
  ylab("Lactonase activity (U/L)") +
  theme_classic() +
  theme(axis.line = element_line(colour = "black", size = 0.5),
```

```
        panel.background = element_rect(fill = "white"),
        panel.grid.major = element_blank(),
        panel.grid.minor = element_blank(),
        axis.text = element_text(size = 12),
        axis.text.x = element_blank(),
        axis.ticks.x = element_blank(),
        axis.title = element_text(size = 14, face = "bold"),
        legend.position =  "none",
        axis.ticks = element_line(colour = "black", size = 0.5),
        aspect.ratio = 2)
ggsave(filename = "Raw plots/Obesity/Lactonact.pdf",
       plot = plot,
       device = "pdf",
       height = 3,
       width = 3)
```

Is PON1 concentration and activity dependent from concomitant diseases?

```
# Select the relevant variables
dependent_vars <- c("PON1c", "ARE_act_2022", "adj_Paraxonase", "adj_La
ctonase")
independent_vars <- c("T2DM", "HT", "DLP", "Metabolic_syn")
dependence_test <- c(dependent_vars, independent_vars)
Db_dependence_test <- Db[, dependence_test]

# Fit a linear regression model for each dependent variable
models <- lapply(dependent_vars, function(var) {
  lm(as.formula(paste(var, "~.", sep = "")), data = Db_dependence_test
)
})

# Extract the p-values from the regression models and put them in a ta
ble
p_values <- sapply(models, function(model) {
  summary(model)$coefficients[2, 4]
})

suppl_table_3 <- data.frame(variable = dependent_vars, p_value = p_val
ues)

print(suppl_table_3)

# PON1c
T2DM_PON1c <- Db %>%
  filter(!is.na(T2DM)) %>%
  ggplot(aes(x = Group, y = PON1c, fill = factor(T2DM))) +
  geom_boxplot(outlier.shape = NA) +
  scale_fill_manual(values = c("#41a8d1", "#e3596d")) +
  labs(x = "Group", y = "PON1 concentration (mg/L)") +
  theme_classic() +
  theme(
    axis.line = element_line(color = "black"),
    axis.text = element_text(size = 12),
    axis.title = element_text(size = 14, face = "bold"),
```

```
    legend.position = "none",
    aspect.ratio = 1.3/1) +
  ylim(0, 300)

p_value <- Db %>%
  filter(Group == "Obese") %>%
  wilcox_test(PON1c ~ T2DM) %>%
  pull(p)
cat("P-value PON1c (when participants suffer or not from T2DM and obes
ity) =", p_value, "\n")

p_value <- Db %>%
  filter(Group == "Control") %>%
  wilcox_test(PON1c ~ T2DM) %>%
  pull(p)

cat("P-value PON1c (when participants suffer or not from T2DM without
obesity) =", p_value, "\n")

ggsave(filename = "Raw plots/Obesity/T2DM_pon1c.pdf",
       plot = T2DM_PON1c,
       device = "pdf",
       height = 3,
       width = 3)

# ARE act
T2DM_ARE <- ggplot(Db, aes(x = Group, y = ARE_act_2022, fill = factor(
T2DM))) +
  geom_boxplot(outlier.shape = NA) +
  scale_fill_manual(values = c("#41a8d1", "#e3596d")) +
  labs(x = "Group", y = "Arylesterease activity (U/L)") +
  theme_classic() +
  theme(
    axis.line = element_line(color = "black"),
    axis.text = element_text(size = 12),
    axis.title = element_text(size = 14, face = "bold"),
    legend.position = "none",
    legend.text = element_text(size = 12),
    legend.title = element_blank(),
    aspect.ratio = 1.3/1
  )

p_value <- Db %>%
  filter(Group == "Obese") %>%
  wilcox_test(ARE_act_2022 ~ T2DM) %>%
  pull(p)
cat("P-value arylesterase activity (when participants suffer or not fr
om T2DM and obesity) =", p_value, "\n")

p_value <- Db %>%
  filter(Group == "Control") %>%
  wilcox_test(ARE_act_2022 ~ T2DM) %>%
  pull(p)
```

```
cat("P-value arylesterase activity (when participants suffer or not fr
om T2DM without obesity) =", p_value, "\n")

ggsave(filename = "Raw plots/Obesity/T2DM_AREact.pdf",
       plot = T2DM_ARE,
       device = "pdf",
       height = 3,
       width = 3)

# PARX act
T2DM_Parax <- Db %>%
  filter(!is.na(T2DM)) %>%
  ggplot(aes(x = Group, y = adj_Paraxonase, fill = factor(T2DM))) +
  geom_boxplot(outlier.shape = NA) +
  scale_fill_manual(values = c("#41a8d1", "#e3596d")) +
  labs(x = "Group", y = "Paraoxonase activity (U/L)") +
  theme_classic() +
  theme(
    axis.line = element_line(color = "black"),
    axis.text = element_text(size = 12),
    axis.title = element_text(size = 14, face = "bold"),
    legend.position = "none",
    aspect.ratio = 1.3/1) +
  ylim(0, 600)

p_value <- Db %>%
  filter(Group == "Obese") %>%
  wilcox_test(adj_Paraxonase ~ T2DM) %>%
  pull(p)
cat("P-value Paraoxonase activity (when participants suffer or not fro
m T2DM and obesity) =", p_value, "\n")

p_value <- Db %>%
  filter(Group == "Control") %>%
  wilcox_test(adj_Paraxonase ~ T2DM) %>%
  pull(p)

cat("P-value Paraoxonase activity (when participants suffer or not fro
m T2DM without obesity) =", p_value, "\n")

ggsave(filename = "Raw plots/Obesity/T2DM_parax.pdf",
       plot = T2DM_Parax,
       device = "pdf",
       height = 3,
       width = 3)

# LAC act
T2DM_Lacton <- Db %>%
  filter(!is.na(T2DM)) %>%
  ggplot(aes(x = Group, y = adj_Lactonase, fill = factor(T2DM))) +
  geom_boxplot(outlier.shape = NA) +
  scale_fill_manual(values = c("#41a8d1", "#e3596d")) +
  labs(x = "Group", y = "Lactonase activity (U/L)") +
  theme_classic() +
```

```
  theme(
    axis.line = element_line(color = "black"),
    axis.text = element_text(size = 12),
    axis.title = element_text(size = 14, face = "bold"),
    legend.position = "none",
    aspect.ratio = 1.3/1) +
  ylim(0, 15)

p_value <- Db %>%
  filter(Group == "Obese") %>%
  wilcox_test(adj_Lactonase ~ T2DM) %>%
  pull(p)
cat("P-value Lactonase activity (when participants suffer or not from
T2DM and obesity) =", p_value, "\n")

p_value <- Db %>%
  filter(Group == "Control") %>%
  wilcox_test(adj_Lactonase ~ T2DM) %>%
  pull(p)

cat("P-value Lactonase activity (when participants suffer or not from
T2DM without obesity) =", p_value, "\n")

ggsave(filename = "Raw plots/Obesity/T2DM_lacton.pdf",
       plot = T2DM_Lacton,
       device = "pdf",
       height = 3,
       width = 3)

# PON1c
HT_PON1c <- Db %>%
  filter(!is.na(HT)) %>%
  ggplot(aes(x = Group, y = PON1c, fill = factor(HT))) +
  geom_boxplot(outlier.shape = NA) +
  scale_fill_manual(values = c("#41a8d1", "#e3596d")) +
  labs(x = "Group", y = "PON1 concentration (mg/L)") +
  theme_classic() +
  theme(
    axis.line = element_line(color = "black"),
    axis.text = element_text(size = 12),
    axis.title = element_text(size = 14, face = "bold"),
    legend.position = "none",
    aspect.ratio = 1.3/1) +
  ylim(0, 300)

p_value <- Db %>%
  filter(Group == "Obese") %>%
  wilcox_test(PON1c ~ HT) %>%
  pull(p)
cat("P-value (when participants suffer or not from HT and obesity) =",
p_value, "\n")

p_value <- Db %>%
  filter(Group == "Control") %>%
```

```
  wilcox_test(PON1c ~ HT) %>%
  pull(p)

cat("P-value (when participants suffer or not from HT without obesity)
=", p_value, "\n")

ggsave(filename = "Raw plots/Obesity/HT_pon1c.pdf",
       plot = HT_PON1c,
       device = "pdf",
       height = 3,
       width = 3)

# ARE act
HT_ARE <- ggplot(Db, aes(x = Group, y = ARE_act_2022, fill = factor(HT
))) +
  geom_boxplot(outlier.shape = NA) +
  scale_fill_manual(values = c("#41a8d1", "#e3596d")) +
  labs(x = "Group", y = "Arylesterease activity (U/L)") +
  theme_classic() +
  theme(
    axis.line = element_line(color = "black"),
    axis.text = element_text(size = 12),
    axis.title = element_text(size = 14, face = "bold"),
    legend.position = "none",
    legend.text = element_text(size = 12),
    legend.title = element_blank(),
    aspect.ratio = 1.3/1
  )

p_value <- Db %>%
  filter(Group == "Obese") %>%
  wilcox_test(ARE_act_2022 ~ HT) %>%
  pull(p)
cat("P-value (when participants suffer or not from HT and obesity) =",
p_value, "\n")

p_value <- Db %>%
  filter(Group == "Control") %>%
  wilcox_test(ARE_act_2022 ~ HT) %>%
  pull(p)

cat("P-value (when participants suffer or not from HT without obesity)
=", p_value, "\n")

ggsave(filename = "Raw plots/Obesity/HT_are.pdf",
       plot = HT_ARE,
       device = "pdf",
       height = 3,
       width = 3)

# PARX act
HT_Parax <- Db %>%
  filter(!is.na(HT)) %>%
  ggplot(aes(x = Group, y = adj_Paraxonase, fill = factor(HT))) +
```

```
  geom_boxplot(outlier.shape = NA) +
  scale_fill_manual(values = c("#41a8d1", "#e3596d")) +
  labs(x = "Group", y = "Paraxonase activity (U/L)") +
  theme_classic() +
  theme(
    axis.line = element_line(color = "black"),
    axis.text = element_text(size = 12),
    axis.title = element_text(size = 14, face = "bold"),
    legend.position = "none",
    aspect.ratio = 1.3/1) +
  ylim(0, 600)

p_value <- Db %>%
  filter(Group == "Obese") %>%
  wilcox_test(adj_Paraxonase ~ HT) %>%
  pull(p)
cat("P-value (when participants suffer or not from HT and obesity) =",
p_value, "\n")

p_value <- Db %>%
  filter(Group == "Control") %>%
  wilcox_test(adj_Paraxonase ~ HT) %>%
  pull(p)

cat("P-value (when participants suffer or not from HT without obesity)
=", p_value, "\n")

ggsave(filename = "Raw plots/Obesity/HT_Parax.pdf",
       plot = HT_Parax,
       device = "pdf",
       height = 3,
       width = 3)

# LAC act
HT_Lacton <- Db %>%
  filter(!is.na(HT)) %>%
  ggplot(aes(x = Group, y = adj_Lactonase, fill = factor(HT))) +
  geom_boxplot(outlier.shape = NA) +
  scale_fill_manual(values = c("#41a8d1", "#e3596d")) +
  labs(x = "Group", y = "Lactonase activity (U/L)") +
  theme_classic() +
  theme(
    axis.line = element_line(color = "black"),
    axis.text = element_text(size = 12),
    axis.title = element_text(size = 14, face = "bold"),
    legend.position = "none",
    aspect.ratio = 1.3/1) +
  ylim(0, 15)

p_value <- Db %>%
  filter(Group == "Obese") %>%
  wilcox_test(adj_Lactonase ~ HT) %>%
  pull(p)
cat("P-value (when participants suffer or not from HT and obesity) =",
```

```
p_value, "\n")

p_value <- Db %>%
  filter(Group == "Control") %>%
  wilcox_test(adj_Lactonase ~ HT) %>%
  pull(p)

cat("P-value (when participants suffer or not from HT without obesity)
=", p_value, "\n")

ggsave(filename = "Raw plots/Obesity/HT_lacton.pdf",
       plot = HT_Lacton,
       device = "pdf",
       height = 3,
       width = 3)

# PON1c
DLP_PON1c <- Db %>%
  filter(!is.na(DLP)) %>%
  ggplot(aes(x = Group, y = PON1c, fill = factor(DLP))) +
  geom_boxplot(outlier.shape = NA) +
  scale_fill_manual(values = c("#41a8d1", "#e3596d")) +
  labs(x = "Group", y = "PON1 concentration (mg/L)") +
  theme_classic() +
  theme(
    axis.line = element_line(color = "black"),
    axis.text = element_text(size = 12),
    axis.title = element_text(size = 14, face = "bold"),
    legend.position = "none",
    aspect.ratio = 1.3/1) +
  ylim(0, 300)

p_value <- Db %>%
  filter(Group == "Obese") %>%
  wilcox_test(PON1c ~ DLP) %>%
  pull(p)
cat("P-value (when participants suffer or not from DLP and obesity) ="
, p_value, "\n")

p_value <- Db %>%
  filter(Group == "Control") %>%
  wilcox_test(PON1c ~ DLP) %>%
  pull(p)

cat("P-value (when participants suffer or not from DLP without obesity
) =", p_value, "\n")

ggsave(filename = "Raw plots/Obesity/DLP_pon1c.pdf",
       plot = DLP_PON1c,
       device = "pdf",
       height = 3,
       width = 3)

# ARE act
DLP_ARE <- ggplot(Db, aes(x = Group, y = ARE_act_2022, fill = factor(D
```

```
LP))) +
  geom_boxplot(outlier.shape = NA) +
  scale_fill_manual(values = c("#41a8d1", "#e3596d")) +
  labs(x = "Group", y = "Arylesterease activity (U/L)") +
  theme_classic() +
  theme(
    axis.line = element_line(color = "black"),
    axis.text = element_text(size = 12),
    axis.title = element_text(size = 14, face = "bold"),
    legend.position = "none",
    legend.text = element_text(size = 12),
    legend.title = element_blank(),
    aspect.ratio = 1.3/1
  )

p_value <- Db %>%
  filter(Group == "Obese") %>%
  wilcox_test(ARE_act_2022 ~ DLP) %>%
  pull(p)
cat("P-value (when participants suffer or not from DLP and obesity) ="
, p_value, "\n")

p_value <- Db %>%
  filter(Group == "Control") %>%
  wilcox_test(ARE_act_2022 ~ DLP) %>%
  pull(p)

cat("P-value (when participants suffer or not from DLP without obesity
) =", p_value, "\n")

ggsave(filename = "Raw plots/Obesity/DLP_are.pdf",
       plot = DLP_ARE,
       device = "pdf",
       height = 3,
       width = 3)

# PARX act
DLP_Parax <- Db %>%
  filter(!is.na(DLP)) %>%
  ggplot(aes(x = Group, y = adj_Paraxonase, fill = factor(DLP))) +
  geom_boxplot(outlier.shape = NA) +
  scale_fill_manual(values = c("#41a8d1", "#e3596d")) +
  labs(x = "Group", y = "Paraoxonasae activity (U/L)") +
  theme_classic() +
  theme(
    axis.line = element_line(color = "black"),
    axis.text = element_text(size = 12),
    axis.title = element_text(size = 14, face = "bold"),
    legend.position = "none",
    aspect.ratio = 1.3/1) +
  ylim(0, 600)

p_value <- Db %>%
  filter(Group == "Obese") %>%
```

```
  wilcox_test(adj_Paraxonase ~ DLP) %>%
  pull(p)
cat("P-value (when participants suffer or not from DLP and obesity) ="
, p_value, "\n")

p_value <- Db %>%
  filter(Group == "Control") %>%
  wilcox_test(adj_Paraxonase ~ DLP) %>%
  pull(p)

cat("P-value (when participants suffer or not from DLP without obesity
) =", p_value, "\n")

ggsave(filename = "Raw plots/Obesity/DLP_parax.pdf",
       plot = DLP_Parax,
       device = "pdf",
       height = 3,
       width = 3)

# LAC act
DLP_Lacton <- Db %>%
  filter(!is.na(DLP)) %>%
  ggplot(aes(x = Group, y = adj_Lactonase, fill = factor(DLP))) +
  geom_boxplot(outlier.shape = NA) +
  scale_fill_manual(values = c("#41a8d1", "#e3596d")) +
  labs(x = "Group", y = "Lactonase activity (U/L)") +
  theme_classic() +
  theme(
    axis.line = element_line(color = "black"),
    axis.text = element_text(size = 12),
    axis.title = element_text(size = 14, face = "bold"),
    legend.position = "none",
    aspect.ratio = 1.3/1) +
  ylim(0, 15)

p_value <- Db %>%
  filter(Group == "Obese") %>%
  wilcox_test(adj_Lactonase ~ DLP) %>%
  pull(p)
cat("P-value (when participants suffer or not from DLP and obesity) ="
, p_value, "\n")

p_value <- Db %>%
  filter(Group == "Control") %>%
  wilcox_test(adj_Lactonase ~ DLP) %>%
  pull(p)

cat("P-value (when participants suffer or not from DLP without obesity
) =", p_value, "\n")

ggsave(filename = "Raw plots/Obesity/DLP_lacton.pdf",
       plot = DLP_Lacton,
       device = "pdf",
```

```
       height = 3,
       width = 3)

# PON1c
Db_filt <- Db %>%
  filter(complete.cases(PON1c, HDL))
cor_plot_PON1c <- ggscatter(Db_filt, x = "HDL", y = "PON1c",
                            add = "reg.line", conf.int = TRUE,
                            cor.coef = TRUE, cor.method = "spearman",
                            title = "Correlation between HDL cholester
ol and PON1 concentration",
                            xlab = "HDL chol (mmol/L)", ylab = "PON1 c
oncentration (mg/L)",
                            legend.title = "none") +
  theme_classic()
  theme(
    axis.line = element_line(color = "black"),
    axis.text = element_text(size = 12),
    axis.title = element_text(size = 12, face = "bold"),
    aspect.ratio = 1/1
  )

ggsave(filename = "Raw plots/Obesity/Corr2HDL_PON1c.pdf",
       plot = cor_plot_PON1c,
       device = "pdf",
       height = 2.5,
       width = 2.5)

# ARE act
Db_filt <- Db %>% filter(Db$HDL<4)
Db_filt <- Db_filt %>%
  filter(complete.cases(ARE_act_2022, HDL))
cor_plot_ARE <- ggscatter(Db_filt, x = "HDL", y = "ARE_act_2022",
                            add = "reg.line", conf.int = TRUE,
                            cor.coef = TRUE, cor.method = "spearman",
                            title = "Correlation between HDL cholester
ol and Arylesterase activity",
                            xlab = "HDL chol (mmol/L)", ylab = "Aryles
terase activity (U/L)",
                            legend.title = "none") +
  theme_classic() +
  theme(
    axis.line = element_line(color = "black"),
    axis.text = element_text(size = 12),
    axis.title = element_text(size = 12, face = "bold"),
    aspect.ratio = 1/1
  )

ggsave(filename = "Raw plots/Obesity/Corr2HDL_ARE.pdf",
       plot = cor_plot_ARE,
       device = "pdf",
       height = 2.5,
       width = 2.5)
```

```
# PARX act
Db_filt <- Db %>%
  filter(complete.cases(adj_Paraxonase, HDL))
cor_plot_Parax <- ggscatter(Db_filt, x = "HDL", y = "adj_Paraxonase",
                            add = "reg.line", conf.int = TRUE,
                            cor.coef = TRUE, cor.method = "spearman",
                            title = "Correlation between HDL cholester
ol and Paraxonase activity",
                            xlab = "HDL chol (mmol/L)", ylab = "Paraxo
nase activity (U/L)",
                            legend.title = "none") +
  theme_classic() +
  theme(
    axis.line = element_line(color = "black"),
    axis.text = element_text(size = 12),
    axis.title = element_text(size = 12, face = "bold"),
    aspect.ratio = 1/1
  )

ggsave(filename = "Raw plots/Obesity/Corr2HDL_Parax.pdf",
       plot = cor_plot_Parax,
       device = "pdf",
       height = 2.5,
       width = 2.5)

# LAC act
Db_filt <- Db %>%
  filter(complete.cases(adj_Lactonase, HDL))
cor_plot_Lacton <- ggscatter(Db_filt, x = "HDL", y = "adj_Lactonase",
                            add = "reg.line", conf.int = TRUE,
                            cor.coef = TRUE, cor.method = "spearman",
                            title = "Correlation between HDL cholester
ol and Lactonase activity",
                            xlab = "HDL chol (mmol/L)", ylab = "Lacton
ase activity (U/L)",
                            legend.title = "none") +
  theme_classic() +
  theme(
    axis.line = element_line(color = "black"),
    axis.text = element_text(size = 12),
    axis.title = element_text(size = 12, face = "bold"),
    aspect.ratio = 1/1
  )

ggsave(filename = "Raw plots/Obesity/Corr2HDL_Lacton.pdf",
       plot = cor_plot_Lacton,
       device = "pdf",
       height = 2.5,
       width = 2.5)

# PON1c
Stat_PON1c <- Db %>%
    filter(Group == "Obese") %>%
    filter(!is.na(Statins)) %>%
```

```
  ggplot(aes(x = Group, y = PON1c, fill = factor(Statins))) +
  geom_boxplot(outlier.shape = NA) +
  scale_fill_manual(values = c("#41a8d1", "#e3596d")) +
  labs(x = "Group", y = "PON1 concentration (mg/L)") +
  theme_classic() +
  theme(
    axis.line = element_line(color = "black"),
    axis.text = element_text(size = 12),
    axis.title = element_text(size = 14, face = "bold"),
    legend.position = "none",
    aspect.ratio = 1.3/0.5) +
  ylim(0, 300)

p_value <- Db %>%
  filter(Group == "Obese") %>%
  wilcox_test(PON1c ~ Statins) %>%
  pull(p)
cat("P-value (when participants take or not Statins) =", p_value, "\n"
)

ggsave(filename = "Raw plots/Obesity/statins_pon1c.pdf",
       plot = Stat_PON1c,
       device = "pdf",
       height = 3,
       width = 3)

# ARE act
Stat_ARE <- Db %>%
    filter(Group == "Obese") %>%
    filter(!is.na(Statins)) %>%
  ggplot(aes(x = Group, y = ARE_act_2022, fill = factor(Statins))) +
  geom_boxplot(outlier.shape = NA) +
  scale_fill_manual(values = c("#41a8d1", "#e3596d")) +
  labs(x = "Group", y = "Arylesterease activity (U/L)") +
  theme_classic() +
  theme(
    axis.line = element_line(color = "black"),
    axis.text = element_text(size = 12),
    axis.title = element_text(size = 14, face = "bold"),
    legend.position = "none",
    legend.text = element_text(size = 12),
    legend.title = element_blank(),
    aspect.ratio = 1.3/0.5
  )

p_value <- Db %>%
  filter(Group == "Obese") %>%
  wilcox_test(ARE_act_2022 ~ Statins) %>%
  pull(p)
cat("P-value (when participants take or not Statins) =", p_value, "\n"
)

ggsave(filename = "Raw plots/Obesity/statins_are.pdf",
       plot = Stat_ARE,
```

```
       device = "pdf",
       height = 3,
       width = 3)

# PARX act
Stat_Parax <- Db %>%
    filter(Group == "Obese") %>%
    filter(!is.na(Statins)) %>%
  ggplot(aes(x = Group, y = adj_Paraxonase, fill = factor(Statins))) +
  geom_boxplot(outlier.shape = NA) +
  scale_fill_manual(values = c("#41a8d1", "#e3596d")) +
  labs(x = "Group", y = "Paraoxonase activity (U/L)") +
  theme_classic() +
  theme(
    axis.line = element_line(color = "black"),
    axis.text = element_text(size = 12),
    axis.title = element_text(size = 14, face = "bold"),
    legend.position = "none",
    aspect.ratio = 1.3/0.5) +
  ylim(0, 600)

p_value <- Db %>%
  filter(Group == "Obese") %>%
  wilcox_test(adj_Paraxonase ~ Statins) %>%
  pull(p)
cat("P-value (when participants take or not Statins) =", p_value, "\n"
)

ggsave(filename = "Raw plots/Obesity/statins_parax.pdf",
       plot = Stat_Parax,
       device = "pdf",
       height = 3,
       width = 3)

# LAC act
Stat_Lacton <- Db %>%
    filter(Group == "Obese") %>%
    filter(!is.na(Statins)) %>%
  ggplot(aes(x = Group, y = adj_Lactonase, fill = factor(Statins))) +
  geom_boxplot(outlier.shape = NA) +
  scale_fill_manual(values = c("#41a8d1", "#e3596d")) +
  labs(x = "Group", y = "Lactonase activity (U/L)") +
  theme_classic() +
  theme(
    axis.line = element_line(color = "black"),
    axis.text = element_text(size = 12),
    axis.title = element_text(size = 14, face = "bold"),
    legend.position = "none",
    aspect.ratio = 1.3/0.5) +
  ylim(0, 15)

p_value <- Db %>%
  filter(Group == "Obese") %>%
  wilcox_test(adj_Lactonase ~ Statins) %>%
```

```
  pull(p)
cat("P-value (when participants take or not statins) =", p_value, "\n"
)

ggsave(filename = "Raw plots/Obesity/statins_lacton.pdf",
       plot = Stat_Lacton,
       device = "pdf",
       height = 3,
       width = 3)

# PON1c
Metabolic_syn_PON1c <- Db %>%
  filter(!is.na(Metabolic_syn)) %>%
  ggplot(aes(x = Group, y = PON1c, fill = factor(Metabolic_syn))) +
  geom_boxplot(outlier.shape = NA) +
  scale_fill_manual(values = c("#41a8d1", "#e3596d")) +
  labs(x = "Group", y = "PON1 concentration (mg/L)") +
  theme_classic() +
  theme(
    axis.line = element_line(color = "black"),
    axis.text = element_text(size = 12),
    axis.title = element_text(size = 14, face = "bold"),
    legend.position = "none",
    aspect.ratio = 1.3/1) +
  ylim(0, 300)

p_value <- Db %>%
  filter(Group == "Obese") %>%
  wilcox_test(PON1c ~ Metabolic_syn) %>%
  pull(p)
cat("P-value PON1 c (when participants suffer or not from Metabolic_sy
n and obesity) =", p_value, "\n")

p_value <- Db %>%
  filter(Group == "Control") %>%
  wilcox_test(PON1c ~ Metabolic_syn) %>%
  pull(p)

cat("P-value PON1 c (when participants suffer or not from Metabolic_sy
n without obesity) =", p_value, "\n")

ggsave(filename = "Raw plots/Obesity/MetS_pon1c.pdf",
       plot = Metabolic_syn_PON1c,
       device = "pdf",
       height = 3,
       width = 3)

# ARE act
Metabolic_syn_ARE <- ggplot(Db, aes(x = Group, y = ARE_act_2022, fill
= factor(HT))) +
  geom_boxplot(outlier.shape = NA) +
  scale_fill_manual(values = c("#41a8d1", "#e3596d")) +
  labs(x = "Group", y = "Arylesterease activity (U/L)") +
  theme_classic() +
```

```
  theme(
    axis.line = element_line(color = "black"),
    axis.text = element_text(size = 12),
    axis.title = element_text(size = 14, face = "bold"),
    legend.position = "none",
    legend.text = element_text(size = 12),
    legend.title = element_blank(),
    aspect.ratio = 1.3/1
  )

p_value <- Db %>%
  filter(Group == "Obese") %>%
  wilcox_test(ARE_act_2022 ~ Metabolic_syn) %>%
  pull(p)
cat("P-value ARE (when participants suffer or not from Metabolic_syn a
nd obesity) =", p_value, "\n")

p_value <- Db %>%
  filter(Group == "Control") %>%
  wilcox_test(ARE_act_2022 ~ Metabolic_syn) %>%
  pull(p)

cat("P-value ARE (when participants suffer or not from Metabolic_syn w
ithout obesity) =", p_value, "\n")

ggsave(filename = "Raw plots/Obesity/MetS_are.pdf",
       plot = Metabolic_syn_ARE,
       device = "pdf",
       height = 3,
       width = 3)

# PARX act
Metabolic_syn_Parax <- Db %>%
  filter(!is.na(Metabolic_syn)) %>%
  ggplot(aes(x = Group, y = adj_Paraxonase, fill = factor(Metabolic_sy
n))) +
  geom_boxplot(outlier.shape = NA) +
  scale_fill_manual(values = c("#41a8d1", "#e3596d")) +
  labs(x = "Group", y = "Paraoxonasae activity (U/L)") +
  theme_classic() +
  theme(
    axis.line = element_line(color = "black"),
    axis.text = element_text(size = 12),
    axis.title = element_text(size = 14, face = "bold"),
    legend.position = "none",
    aspect.ratio = 1.3/1) +
  ylim(0, 600)

p_value <- Db %>%
  filter(Group == "Obese") %>%
  wilcox_test(adj_Paraxonase ~ Metabolic_syn) %>%
  pull(p)
cat("P-value Paraoxonase act (when participants suffer or not from Met
abolic_syn and obesity) =", p_value, "\n")
```

```
p_value <- Db %>%
  filter(Group == "Control") %>%
  wilcox_test(adj_Paraxonase ~ Metabolic_syn) %>%
  pull(p)

cat("P-value Paraoxonase act (when participants suffer or not from Met
abolic_syn without obesity) =", p_value, "\n")

ggsave(filename = "Raw plots/Obesity/MetS_parax.pdf",
       plot = Metabolic_syn_Parax,
       device = "pdf",
       height = 3,
       width = 3)

# LAC act
Metabolic_syn_Lacton <- Db %>%
  filter(!is.na(Metabolic_syn)) %>%
  ggplot(aes(x = Group, y = adj_Lactonase, fill = factor(Metabolic_syn
))) +
  geom_boxplot(outlier.shape = NA) +
  scale_fill_manual(values = c("#41a8d1", "#e3596d")) +
  labs(x = "Group", y = "Lactonase activity (U/L)") +
  theme_classic() +
  theme(
    axis.line = element_line(color = "black"),
    axis.text = element_text(size = 12),
    axis.title = element_text(size = 14, face = "bold"),
    legend.position = "none",
    aspect.ratio = 1.3/1) +
  ylim(0, 15)

p_value <- Db %>%
  filter(Group == "Obese") %>%
  wilcox_test(adj_Lactonase ~ Metabolic_syn) %>%
  pull(p)
cat("P-value Lactonase act (when participants suffer or not from Metab
olic_syn and obesity) =", p_value, "\n")

p_value <- Db %>%
  filter(Group == "Control") %>%
  wilcox_test(adj_Lactonase ~ Metabolic_syn) %>%
  pull(p)

cat("P-value Lactonase act (when participants suffer or not from Metab
olic_syn without obesity) =", p_value, "\n")

ggsave(filename = "Raw plots/Obesity/MetS_Lacton.pdf",
       plot = Metabolic_syn_Lacton,
       device = "pdf",
       height = 3,
       width = 3)
```

BMI, waist circumference, and PON1

```
# PON1c
Db_filt <- Db %>%
  filter(complete.cases(PON1c, BMI))
cor_plot_PON1c <- ggscatter(Db_filt, x = "BMI", y = "PON1c",
                            add = "reg.line", conf.int = TRUE,
                            cor.coef = TRUE, cor.method = "spearman",
                            title = "Correlation between BMI and PON1
concentration",
                            xlab = "BMI (kg/m2)", ylab = "PON1 concent
ration (mg/L)",
                            legend.title = "none") +
  theme_classic() +
  theme(
    axis.line = element_line(color = "black"),
    axis.text = element_text(size = 12),
    axis.title = element_text(size = 12, face = "bold"),
    aspect.ratio = 1/1
  )

# ARE act
Db_filt <- Db %>%
  filter(complete.cases(ARE_act_2022, BMI))
cor_plot_ARE <- ggscatter(Db_filt, x = "BMI", y = "ARE_act_2022",
                            add = "reg.line", conf.int = TRUE,
                            cor.coef = TRUE, cor.method = "spearman",
                            title = "Correlation between BMI and aryle
sterase activity",
                            xlab = "BMI (kg/m2)", ylab = "Arylesterase
activity (U/L)",
                            legend.title = "none") +
  theme_classic() +
  theme(
    axis.line = element_line(color = "black"),
    axis.text = element_text(size = 12),
    axis.title = element_text(size = 12, face = "bold"),
    aspect.ratio = 1/1
  )

# PARX act
Db_filt <- Db %>%
  filter(complete.cases(adj_Paraxonase, BMI))
cor_plot_Parax <- ggscatter(Db_filt, x = "BMI", y = "adj_Paraxonase",
                            add = "reg.line", conf.int = TRUE,
                            cor.coef = TRUE, cor.method = "spearman",
                            title = "Correlation between BMI and Parax
onase activity",
                            xlab = "BMI (kg/m2)", ylab = "Paraxonase a
ctivity (U/L)",
                            legend.title = "none") +
  theme_classic() +
  theme(
    axis.line = element_line(color = "black"),
    axis.text = element_text(size = 12),
```

```
    axis.title = element_text(size = 12, face = "bold"),
    aspect.ratio = 1/1
  )

# LAC act
Db_filt <- Db %>%
  filter(complete.cases(adj_Lactonase, BMI))
cor_plot_Lacto <- ggscatter(Db_filt, x = "BMI", y = "adj_Lactonase",
                            add = "reg.line", conf.int = TRUE,
                            cor.coef = TRUE, cor.method = "spearman",
                            title = "Correlation between BMI and Lacto
nase activity",
                            xlab = "BMI (kg/m2)", ylab = "Lactonase ac
tivity (U/L)",
                            legend.title = "none") +
  theme_classic() +
  theme(
    axis.line = element_line(color = "black"),
    axis.text = element_text(size = 12),
    axis.title = element_text(size = 12, face = "bold"),
    aspect.ratio = 1/1
  )

# Save the plots
ggsave(filename = "Raw plots/Obesity/cor2BMI_PON1c.pdf",
       plot = cor_plot_PON1c,
       device = "pdf",
       height = 2.5,
       width = 2.5)
ggsave(filename = "Raw plots/Obesity/cor2BMI_are.pdf",
       plot = cor_plot_ARE,
       device = "pdf",
       height = 2.5,
       width = 2.5)
ggsave(filename = "Raw plots/Obesity/cor2BMI_parax.pdf",
       plot = cor_plot_Parax,
       device = "pdf",
       height = 2.5,
       width = 2.5)
ggsave(filename = "Raw plots/Obesity/cor2BMI_Lacton.pdf",
       plot = cor_plot_Lacto,
       device = "pdf",
       height = 2.5,
       width = 2.5)

# PON1c
Db_filt <- Db %>%
  filter(complete.cases(PON1c, Pcintura))
cor_plot_PON1c <- ggscatter(Db_filt, x = "Pcintura", y = "PON1c",
                            add = "reg.line", conf.int = TRUE,
                            cor.coef = TRUE, cor.method = "spearman",
                            title = "Correlation between Pcintura and
PON1 concentration",
                            xlab = "Waist circumference (cm)", ylab =
```

```
"PON1 concentration (mg/L)",
                              legend.title = "none") +
  theme_classic() +
  theme(
    axis.line = element_line(color = "black"),
    axis.text = element_text(size = 12),
    axis.title = element_text(size = 12, face = "bold"),
    aspect.ratio = 1/1
  )

# ARE act
Db_filt <- Db %>%
  filter(complete.cases(ARE_act_2022, Pcintura))
cor_plot_ARE <- ggscatter(Db_filt, x = "Pcintura", y = "ARE_act_2022",
                              add = "reg.line", conf.int = TRUE,
                              cor.coef = TRUE, cor.method = "spearman",
                              title = "Correlation between waist circumf
erence and arylesterase activity",
                              xlab = "Waist circumference (cm)", ylab =
"Arylesterase activity (U/L)",
                              legend.title = "none") +
  theme_classic() +
  theme(
    axis.line = element_line(color = "black"),
    axis.text = element_text(size = 12),
    axis.title = element_text(size = 12, face = "bold"),
    aspect.ratio = 1/1
  )

# PARX act
Db_filt <- Db %>%
  filter(complete.cases(adj_Paraxonase, Pcintura))
cor_plot_Parax <- ggscatter(Db_filt, x = "Pcintura", y = "adj_Paraxona
se",
                              add = "reg.line", conf.int = TRUE,
                              cor.coef = TRUE, cor.method = "spearman",
                              title = "Correlation between waist circumf
erence and Paraxonase activity",
                              xlab = "Waist circumference (cm)", ylab =
"Paraxonase activity (U/L)",
                              legend.title = "none") +
  theme_classic() +
  theme(
    axis.line = element_line(color = "black"),
    axis.text = element_text(size = 12),
    axis.title = element_text(size = 12, face = "bold"),
    aspect.ratio = 1/1
  )

# LAC act
Db_filt <- Db %>%
  filter(complete.cases(adj_Lactonase, Pcintura))
cor_plot_Lacto <- ggscatter(Db_filt, x = "Pcintura", y = "adj_Lactonas
e",
```

```
                            add = "reg.line", conf.int = TRUE,
                            cor.coef = TRUE, cor.method = "spearman",
                            title = "Correlation between waist circumf
erence and Lactonase activity",
                            xlab = "Waist circumference (cm)", ylab =
"Lactonase activity (U/L)",
                            legend.title = "none") +
  theme_classic() +
  theme(
    axis.line = element_line(color = "black"),
    axis.text = element_text(size = 12),
    axis.title = element_text(size = 12, face = "bold"),
    aspect.ratio = 1/1
  )

# Save the plots
ggsave(filename = "Raw plots/Obesity/cor2Pcin_PON1c.pdf",
       plot = cor_plot_PON1c,
       device = "pdf",
       height = 2.5,
       width = 2.5)
ggsave(filename = "Raw plots/Obesity/cor2Pcin_are.pdf",
       plot = cor_plot_ARE,
       device = "pdf",
       height = 2.5,
       width = 2.5)
ggsave(filename = "Raw plots/Obesity/cor2Pcin_parax.pdf",
       plot = cor_plot_Parax,
       device = "pdf",
       height = 2.5,
       width = 2.5)
ggsave(filename = "Raw plots/Obesity/cor2Pcin_Lacton.pdf",
       plot = cor_plot_Lacto,
       device = "pdf",
       height = 2.5,
       width = 2.5)
```

Is hepatic severity related to PON1 concentration and activity?

```
DbNASH <- subset(Db, Db$Group == "Obese")
DbNASH$NAS_qual <- as.factor(DbNASH$NAS_qual)
DbNASH$Steatosis_score <- as.factor(DbNASH$Steatosis_score)
DbNASH$Ballooning <- as.factor(DbNASH$Ballooning)
DbNASH$Inflammation <- as.factor(DbNASH$Inflammation)
table_2 <- CreateTableOne(data = DbNASH, vars = vars, factorVars = cat
vars, strata = "NAS_qual")
print(suppl_table_4, nonnormal = vars, formatOptions = list(big.mark =
","))
```

PON1 and NASH diagnosis

```
PON1c <- DbNASH %>%
  filter(complete.cases(NAS_qual)) %>%
```

```
  ggplot(aes(x = NAS_qual, y = PON1c, fill = NAS_qual)) +
  geom_boxplot(outlier.shape=NA, color = "black", size = 0.5) +
  stat_compare_means(label = "p", tip.length = 0, size = 4) +
  scale_fill_manual(values = c("#fafa6e", "#23aa8f", "#d02a5a")) +
  ylim(0, 300) +
  xlab("") +
  ylab("PON1 concentration (mg/L)") +
  theme_classic() +
  theme(axis.line = element_line(colour = "black", size = 0.5),
        panel.background = element_rect(fill = "white"),
        panel.grid.major = element_blank(),
        panel.grid.minor = element_blank(),
        axis.text = element_text(size = 12),
        axis.text.x = element_blank(),
        axis.ticks.x = element_blank(),
        axis.title = element_text(size = 14, face = "bold"),
        #legend.position =  "none",
        axis.ticks = element_line(colour = "black", size = 0.5),
        aspect.ratio = 2)
ggsave(filename = "Raw plots/Liver/NASqual_pon1c.pdf",
       plot = PON1c,
       device = "pdf",
       height = 3,
       width = 5)

are <- DbNASH %>%   filter(complete.cases(Steatosis_score)) %>%   ggpl
ot(aes(x = NAS_qual, y = ARE_act_2022, fill = NAS_qual)) +
  geom_boxplot(outlier.shape=NA, color = "black", size = 0.5) +
  stat_compare_means(label = "p", tip.length = 0, size = 4) +
  scale_fill_manual(values = c("#fafa6e", "#23aa8f", "#d02a5a")) +
  ylim(0, 300) +
  xlab("") +
  ylab("Arylesterease activity (U/L)") +
  theme_classic() +
  theme(axis.line = element_line(colour = "black", size = 0.5),
        panel.background = element_rect(fill = "white"),
        panel.grid.major = element_blank(),
        panel.grid.minor = element_blank(),
        axis.text = element_text(size = 12),
        axis.text.x = element_blank(),
        axis.ticks.x = element_blank(),
        axis.title = element_text(size = 14, face = "bold"),
        #legend.position =  "none",
        axis.ticks = element_line(colour = "black", size = 0.5),
        aspect.ratio = 2)
ggsave(filename = "Raw plots/Liver/NASqual_are.pdf",
       plot = are,
       device = "pdf",
       height = 3,
       width = 5)

parax <- DbNASH %>%   filter(complete.cases(Steatosis_score)) %>%   gg
plot(aes(x = NAS_qual, y = adj_Paraxonase, fill = NAS_qual)) +
  geom_boxplot(outlier.shape=NA, color = "black", size = 0.5) +
```

```
  stat_compare_means(label = "p", tip.length = 0, size = 4) +
  scale_fill_manual(values = c("#fafa6e", "#23aa8f", "#d02a5a")) +
  ylim(0, 500) +
  xlab("") +
  ylab("Paraoxonasae activity (U/L)") +
  theme_classic() +
  theme(axis.line = element_line(colour = "black", size = 0.5),
        panel.background = element_rect(fill = "white"),
        panel.grid.major = element_blank(),
        panel.grid.minor = element_blank(),
        axis.text = element_text(size = 12),
        axis.text.x = element_blank(),
        axis.ticks.x = element_blank(),
        axis.title = element_text(size = 14, face = "bold"),
        #legend.position =  "none",
        axis.ticks = element_line(colour = "black", size = 0.5),
        aspect.ratio = 2)
ggsave(filename = "Raw plots/Liver/NASqual_parax.pdf",
       plot = parax,
       device = "pdf",
       height = 3,
       width = 5)

lacton <- DbNASH %>%   filter(complete.cases(Steatosis_score)) %>%   g
gplot(aes(x = NAS_qual, y = adj_Lactonase, fill = NAS_qual)) +
  geom_boxplot(outlier.shape=NA, color = "black", size = 0.5) +
  stat_compare_means(label = "p", tip.length = 0, size = 4) +
  scale_fill_manual(values = c("#fafa6e", "#23aa8f", "#d02a5a")) +
  ylim(0, 15) +
  xlab("") +
  ylab("Lactonase activity (U/L)") +
  theme_classic() +
  theme(axis.line = element_line(colour = "black", size = 0.5),
        panel.background = element_rect(fill = "white"),
        panel.grid.major = element_blank(),
        panel.grid.minor = element_blank(),
        axis.text = element_text(size = 12),
        axis.text.x = element_blank(),
        axis.ticks.x = element_blank(),
        axis.title = element_text(size = 14, face = "bold"),
        #legend.position =  "none",
        axis.ticks = element_line(colour = "black", size = 0.5),
        aspect.ratio = 2)
ggsave(filename = "Raw plots/Liver/NASqual_lacton.pdf",
       plot = lacton,
       device = "pdf",
       height = 3,
       width = 5)
```

PON1 and hepatic histological features

```
#Definition of hepatic features as categorical variables
DbNASH$Steatosis_score <- as.factor(DbNASH$Steatosis_score)
DbNASH$Inflammation <- as.factor(DbNASH$Inflammation)
```

```
DbNASH$Ballooning <- as.factor(DbNASH$Ballooning)
DbNASH$Fibrosis <- as.factor(DbNASH$Fibrosis)
```

Steatosis score

```
steat_pon1c <- DbNASH %>%   filter(complete.cases(Steatosis_score)) %>
%   ggplot(aes(x = Steatosis_score, y = PON1c, fill = Steatosis_score)
) +
  geom_boxplot(outlier.shape = NA, color = "black", size = 0.5, na.rm
= TRUE) +
  stat_compare_means(label = "p", tip.length = 0, size = 4) +
  scale_fill_manual(values = c("#8ca6bd", "#f6b02c", "#c73647", "#3133
40")) +
  ylim(0, 300) +
  xlab("") +
  ylab("PON1 concentration (mg/L)") +
  theme_classic() +
  theme(axis.line = element_line(colour = "black", size = 0.5),
        panel.background = element_rect(fill = "white"),
        panel.grid.major = element_blank(),
        panel.grid.minor = element_blank(),
        axis.text = element_text(size = 12),
        axis.text.x = element_blank(),
        axis.ticks.x = element_blank(),
        axis.title = element_text(size = 14, face = "bold"),
        #legend.position =  "none",
        axis.ticks = element_line(colour = "black", size = 0.5),
        aspect.ratio = 2)
ggsave(filename = "Raw plots/Liver/steat_pon1c.pdf",
       plot = steat_pon1c,
       device = "pdf",
       height = 3,
       width = 5)

steat_are <- DbNASH %>%   filter(complete.cases(Steatosis_score)) %>%
ggplot(aes(x = Steatosis_score, y = ARE_act_2022, fill = Steatosis_sco
re)) +
  geom_boxplot(outlier.shape=NA, color = "black", size = 0.5) +
  stat_compare_means(label = "p", tip.length = 0, size = 4) +
  scale_fill_manual(values = c("#8ca6bd", "#f6b02c", "#c73647", "#3133
40")) +
  ylim(0, 300) +
  xlab("") +
  ylab("Arylesterease activity (U/L)") +
  theme_classic() +
  theme(axis.line = element_line(colour = "black", size = 0.5),
        panel.background = element_rect(fill = "white"),
        panel.grid.major = element_blank(),
        panel.grid.minor = element_blank(),
        axis.text = element_text(size = 12),
        axis.text.x = element_blank(),
        axis.ticks.x = element_blank(),
        axis.title = element_text(size = 14, face = "bold"),
        #legend.position =  "none",
        axis.ticks = element_line(colour = "black", size = 0.5),
```

```
        aspect.ratio = 2)
ggsave(filename = "Raw plots/Liver/steat_are.pdf",
       plot = steat_are,
       device = "pdf",
       height = 3,
       width = 5)

steat_parax <- DbNASH %>%   filter(complete.cases(Steatosis_score)) %>
%   ggplot(aes(x = Steatosis_score, y = adj_Paraxonase, fill = Steatos
is_score)) +
  geom_boxplot(outlier.shape=NA, color = "black", size = 0.5) +
  stat_compare_means(label = "p", tip.length = 0, size = 4) +
  scale_fill_manual(values = c("#8ca6bd", "#f6b02c", "#c73647", "#3133
40")) +
  ylim(0, 500) +
  xlab("") +
  ylab("Paraoxonasae activity (U/L)") +
  theme_classic() +
  theme(axis.line = element_line(colour = "black", size = 0.5),
        panel.background = element_rect(fill = "white"),
        panel.grid.major = element_blank(),
        panel.grid.minor = element_blank(),
        axis.text = element_text(size = 12),
        axis.text.x = element_blank(),
        axis.ticks.x = element_blank(),
        axis.title = element_text(size = 14, face = "bold"),
        #legend.position =  "none",
        axis.ticks = element_line(colour = "black", size = 0.5),
        aspect.ratio = 2)
ggsave(filename = "Raw plots/Liver/steat_parax.pdf",
       plot = steat_parax,
       device = "pdf",
       height = 3,
       width = 5)

steat_lacton <- DbNASH %>%   filter(complete.cases(Steatosis_score)) %
>%   ggplot(aes(x = Steatosis_score, y = adj_Lactonase, fill = Steatos
is_score)) +
  geom_boxplot(outlier.shape=NA, color = "black", size = 0.5) +
  stat_compare_means(label = "p", tip.length = 0, size = 4) +
  scale_fill_manual(values = c("#8ca6bd", "#f6b02c", "#c73647", "#3133
40")) +
  ylim(0, 15) +
  xlab("") +
  ylab("Lactonase activity (U/L)") +
  theme_classic() +
  theme(axis.line = element_line(colour = "black", size = 0.5),
        panel.background = element_rect(fill = "white"),
        panel.grid.major = element_blank(),
        panel.grid.minor = element_blank(),
        axis.text = element_text(size = 12),
        axis.text.x = element_blank(),
        axis.ticks.x = element_blank(),
        axis.title = element_text(size = 14, face = "bold"),
```

```
        #legend.position =  "none",
        axis.ticks = element_line(colour = "black", size = 0.5),
        aspect.ratio = 2)
ggsave(filename = "Raw plots/Liver/steat_lacton.pdf",
       plot = steat_lacton,
       device = "pdf",
       height = 3,
       width = 5)
```

Steatosis grade

```
# Filter data and calculate correlations with tie handling
Db_filt <- DbNASH %>%
  filter(complete.cases(Steatosis_grade))

cor_result_PON1c <- cor.test(Db_filt$PON1c, Db_filt$Steatosis_grade, m
ethod = "spearman", exact = FALSE)
cor_result_ARE <- cor.test(Db_filt$ARE_act_2022, Db_filt$Steatosis_gra
de, method = "spearman", exact = FALSE)
cor_result_Parax <- cor.test(Db_filt$adj_Paraxonase, Db_filt$Steatosis
_grade, method = "spearman", exact = FALSE)
cor_result_Lacto <- cor.test(Db_filt$adj_Lactonase, Db_filt$Steatosis_
grade, method = "spearman", exact = FALSE)

# Create correlation plots
cor_plot_PON1c <- ggscatter(Db_filt, x = "Steatosis_grade", y = "PON1c
",
                            add = "reg.line", conf.int = TRUE,
                            cor.coef = TRUE, cor.method = "spearman",
                            title = "Correlation between steatosis gra
de and PON1 concentration",
                            xlab = "Steatosis grade (%)", ylab = "PON1
concentration (mg/L)",
                            legend.title = paste("rho =", round(cor_re
sult_PON1c$estimate, 2),
                                                 "p =", format(cor_res
ult_PON1c$p.value, digits = 4)),
                            legend = "bottom", legend.params = list(co
lor = "red")) +
   theme_classic() +
  theme(
    axis.line = element_line(color = "black"),
    axis.text = element_text(size = 12),
    axis.title = element_text(size = 12, face = "bold"),
    legend.position = "none"
  )

cor_plot_ARE <- ggscatter(Db_filt, x = "Steatosis_grade", y = "ARE_act
_2022",
                          add = "reg.line", conf.int = TRUE,
                          cor.coef = TRUE, cor.method = "spearman",
                          title = "Correlation between steatosis grade
and Arylesterease activity",
                          xlab = "Steatosis grade (%)", ylab = "Aryles
terase activity (U/L)",
```

```
                              legend.title = paste("rho =", round(cor_resu
lt_ARE$estimate, 2),
                                                   "p =", format(cor_resul
t_ARE$p.value, digits = 4)),
                              legend = "bottom", legend.params = list(colo
r = "red")) +
   theme_classic() +
  theme(
    axis.line = element_line(color = "black"),
    axis.text = element_text(size = 12),
    axis.title = element_text(size = 12, face = "bold"),
    legend.position = "none"
  )

cor_plot_Parax <- ggscatter(Db_filt, x = "Steatosis_grade", y = "adj_P
araxonase",
                            add = "reg.line", conf.int = TRUE,
                            cor.coef = TRUE, cor.method = "spearman",
                            title = "Correlation between steatosis gra
de and Paraoxonase activity",
                            xlab = "Steatosis grade (%)", ylab = "Para
oxonasae activity (U/L)",
                            legend.title = paste("rho =", round(cor_re
sult_Parax$estimate, 2),
                                                 "p =", format(cor_res
ult_Parax$p.value, digits = 4)),
                            legend = "bottom", legend.params = list(co
lor = "red")) +
   theme_classic() +
  theme(
    axis.line = element_line(color = "black"),
    axis.text = element_text(size = 12),
    axis.title = element_text(size = 12, face = "bold"),
    legend.position = "none"
  )

cor_plot_Lacto <- ggscatter(Db_filt, x = "Steatosis_grade", y = "adj_L
actonase",
          add = "reg.line", conf.int = TRUE,
          cor.coef = TRUE, cor.method = "spearman",
          title = "Correlation between steatosis grade and adj_Lactona
se activity",
          xlab = "Steatosis grade (%)", ylab = "Lactonase activity (U/
L)",
          legend.title = paste("rho =", round(cor_result_Lacto$estimat
e, 2),
                                             "p =", format(cor_result_Lacto$p.value,
digits = 4)),
          legend = "bottom", legend.params = list(color = "red")) +
  theme_classic() +
  theme(
    axis.line = element_line(color = "black"),
    axis.text = element_text(size = 12),
    axis.title = element_text(size = 12, face = "bold"),
```

```
    legend.position = "none"
  )
# Save the plots
ggsave(filename = "Raw plots/Liver/corSteat_PON1c.pdf",
       plot = cor_plot_PON1c,
       device = "pdf",
       height = 3.2,
       width = 5.2)
ggsave(filename = "Raw plots/Liver/corSteat_are.pdf",
       plot = cor_plot_ARE,
       device = "pdf",
       height = 3.2,
       width = 5.2)
ggsave(filename = "Raw plots/Liver/corSteat_parax.pdf",
       plot = cor_plot_Parax,
       device = "pdf",
       height = 3.2,
       width = 5.2)
ggsave(filename = "Raw plots/Liver/corSteat_Lacton.pdf",
       plot = cor_plot_Lacton,
       device = "pdf",
       height = 3.2,
       width = 5.2)
```

Lobular inflammation

```
infl_pon1c <- DbNASH %>%   filter(complete.cases(Inflammation)) %>%
ggplot(aes(x = Inflammation, y = PON1c, fill = Inflammation)) +
  geom_boxplot(outlier.shape=NA, color = "black", size = 0.5) +
  stat_compare_means(label = "p", tip.length = 0, size = 4) +
  scale_fill_manual(values = c("#FFB500", "#FFDA13", "#5F7CA1", "#2735
50")) +
  ylim(0, 300) +
  xlab("") +
  ylab("PON1 concentration (mg/L)") +
  theme_classic() +
  theme(axis.line = element_line(colour = "black", size = 0.5),
        panel.background = element_rect(fill = "white"),
        panel.grid.major = element_blank(),
        panel.grid.minor = element_blank(),
        axis.text = element_text(size = 12),
        axis.text.x = element_blank(),
        axis.ticks.x = element_blank(),
        axis.title = element_text(size = 14, face = "bold"),
        #legend.position =  "none",
        axis.ticks = element_line(colour = "black", size = 0.5),
        aspect.ratio = 2)
ggsave(filename = "Raw plots/Liver/infl_pon1c.pdf",
       plot = infl_pon1c,
       device = "pdf",
       height = 3,
       width = 5)

infl_are <- DbNASH %>%   filter(complete.cases(Inflammation)) %>%   gg
plot(aes(x = Inflammation, y = ARE_act_2022, fill = Inflammation)) +
```

```
  geom_boxplot(outlier.shape=NA, color = "black", size = 0.5) +
  #geom_point() +
  stat_compare_means(label = "p", tip.length = 0, size = 4) +
  scale_fill_manual(values = c("#FFB500", "#FFDA13", "#5F7CA1", "#2735
50")) +
  ylim(0, 300) +
  xlab("") +
  ylab("Arylesterease activity (U/L)") +
  theme_classic() +
  theme(axis.line = element_line(colour = "black", size = 0.5),
        panel.background = element_rect(fill = "white"),
        panel.grid.major = element_blank(),
        panel.grid.minor = element_blank(),
        axis.text = element_text(size = 12),
        axis.text.x = element_blank(),
        axis.ticks.x = element_blank(),
        axis.title = element_text(size = 14, face = "bold"),
        #legend.position =  "none",
        axis.ticks = element_line(colour = "black", size = 0.5),
        aspect.ratio = 2)
ggsave(filename = "Raw plots/Liver/infl_are.pdf",
       plot = infl_are,
       device = "pdf",
       height = 3,
       width = 5)

infl_parax <- DbNASH %>%   filter(complete.cases(Inflammation)) %>%
ggplot(aes(x = Inflammation, y = adj_Paraxonase, fill = Inflammation))
+
  geom_boxplot(outlier.shape=NA, color = "black", size = 0.5) +
  stat_compare_means(label = "p", tip.length = 0, size = 4) +
  scale_fill_manual(values = c("#FFB500", "#FFDA13", "#5F7CA1", "#2735
50")) +
  ylim(0, 500) +
  xlab("") +
  ylab("Paraoxonasae activity (U/L)") +
  theme_classic() +
  theme(axis.line = element_line(colour = "black", size = 0.5),
        panel.background = element_rect(fill = "white"),
        panel.grid.major = element_blank(),
        panel.grid.minor = element_blank(),
        axis.text = element_text(size = 12),
        axis.text.x = element_blank(),
        axis.ticks.x = element_blank(),
        axis.title = element_text(size = 14, face = "bold"),
        #legend.position =  "none",
        axis.ticks = element_line(colour = "black", size = 0.5),
        aspect.ratio = 2)
ggsave(filename = "Raw plots/Liver/infl_parax.pdf",
       plot = infl_parax,
       device = "pdf",
       height = 3,
       width = 5)
```

```
infl_lacton <- DbNASH %>%   filter(complete.cases(Inflammation)) %>%
ggplot(aes(x = Inflammation, y = adj_Lactonase, fill = Inflammation))
+
  geom_boxplot(outlier.shape=NA, color = "black", size = 0.5) +
  stat_compare_means(label = "p", tip.length = 0, size = 4) +
  scale_fill_manual(values = c("#FFB500", "#FFDA13", "#5F7CA1", "#2735
50")) +
  ylim(0, 15) +
  xlab("") +
  ylab("Lactonase activity (U/L)") +
  theme_classic() +
  theme(axis.line = element_line(colour = "black", size = 0.5),
        panel.background = element_rect(fill = "white"),
        panel.grid.major = element_blank(),
        panel.grid.minor = element_blank(),
        axis.text = element_text(size = 12),
        axis.text.x = element_blank(),
        axis.ticks.x = element_blank(),
        axis.title = element_text(size = 14, face = "bold"),
        #legend.position =  "none",
        axis.ticks = element_line(colour = "black", size = 0.5),
        aspect.ratio = 2)
ggsave(filename = "Raw plots/Liver/infl_lacton.pdf",
       plot = infl_lacton,
       device = "pdf",
       height = 3,
       width = 5)
```

Ballooning

```
ballo_pon1c <- DbNASH %>%   filter(complete.cases(Ballooning)) %>%   g
gplot(aes(x = Ballooning, y = PON1c, fill = Ballooning)) +
  geom_boxplot(outlier.shape=NA, color = "black", size = 0.5) +
  stat_compare_means(label = "p", tip.length = 0, size = 4) +
  scale_fill_manual(values = c("#653040", "#dfdcd7", "#6b9fc6")) +
  ylim(0, 300) +
  xlab("") +
  ylab("PON1 concentration (mg/L)") +
  theme_classic() +
  theme(axis.line = element_line(colour = "black", size = 0.5),
        panel.background = element_rect(fill = "white"),
        panel.grid.major = element_blank(),
        panel.grid.minor = element_blank(),
        axis.text = element_text(size = 12),
        axis.text.x = element_blank(),
        axis.ticks.x = element_blank(),
        axis.title = element_text(size = 14, face = "bold"),
        #legend.position =  "none",
        axis.ticks = element_line(colour = "black", size = 0.5),
        aspect.ratio = 2)
ggsave(filename = "Raw plots/Liver/ballo_pon1c.pdf",
       plot = ballo_pon1c,
       device = "pdf",
       height = 3,
       width = 5)
```

```
ballo_are <- DbNASH %>%   filter(complete.cases(Ballooning)) %>%   ggp
lot(aes(x = Ballooning, y = ARE_act_2022, fill = Ballooning)) +
  geom_boxplot(outlier.shape=NA, color = "black", size = 0.5) +
  #geom_point() +
  stat_compare_means(label = "p", tip.length = 0, size = 4) +
  scale_fill_manual(values = c("#653040", "#dfdcd7", "#6b9fc6")) +
  ylim(0, 300) +
  xlab("") +
  ylab("Arylesterease activity (U/L)") +
  theme_classic() +
  theme(axis.line = element_line(colour = "black", size = 0.5),
        panel.background = element_rect(fill = "white"),
        panel.grid.major = element_blank(),
        panel.grid.minor = element_blank(),
        axis.text = element_text(size = 12),
        axis.text.x = element_blank(),
        axis.ticks.x = element_blank(),
        axis.title = element_text(size = 14, face = "bold"),
        #legend.position =  "none",
        axis.ticks = element_line(colour = "black", size = 0.5),
        aspect.ratio = 2)
ggsave(filename = "Raw plots/Liver/ballo_are.pdf",
       plot = ballo_are,
       device = "pdf",
       height = 3,
       width = 5)

ballo_parax <- DbNASH %>%   filter(complete.cases(Ballooning)) %>%   g
gplot(aes(x = Ballooning, y = adj_Paraxonase, fill = Ballooning)) +
  geom_boxplot(outlier.shape=NA, color = "black", size = 0.5) +
  stat_compare_means(label = "p", tip.length = 0, size = 4) +
  scale_fill_manual(values = c("#653040", "#dfdcd7", "#6b9fc6")) +
  ylim(0, 500) +
  xlab("") +
  ylab("Paraoxonasae activity (U/L)") +
  theme_classic() +
  theme(axis.line = element_line(colour = "black", size = 0.5),
        panel.background = element_rect(fill = "white"),
        panel.grid.major = element_blank(),
        panel.grid.minor = element_blank(),
        axis.text = element_text(size = 12),
        axis.text.x = element_blank(),
        axis.ticks.x = element_blank(),
        axis.title = element_text(size = 14, face = "bold"),
        #legend.position =  "none",
        axis.ticks = element_line(colour = "black", size = 0.5),
        aspect.ratio = 2)
ggsave(filename = "Raw plots/Liver/ballo_parax.pdf",
       plot = ballo_parax,
       device = "pdf",
       height = 3,
       width = 5)
```

```
ballo_lacto <- DbNASH %>%   filter(complete.cases(Ballooning)) %>%   g
gplot(aes(x = Ballooning, y = adj_Lactonase, fill = Ballooning)) +
  geom_boxplot(outlier.shape=NA, color = "black", size = 0.5) +
  stat_compare_means(label = "p", tip.length = 0, size = 4) +
  scale_fill_manual(values = c("#653040", "#dfdcd7", "#6b9fc6")) +
  ylim(0, 15) +
  xlab("") +
  ylab("Lactonase activity (U/L)") +
  theme_classic() +
  theme(axis.line = element_line(colour = "black", size = 0.5),
        panel.background = element_rect(fill = "white"),
        panel.grid.major = element_blank(),
        panel.grid.minor = element_blank(),
        axis.text = element_text(size = 12),
        axis.text.x = element_blank(),
        axis.ticks.x = element_blank(),
        axis.title = element_text(size = 14, face = "bold"),
        #legend.position =  "none",
        axis.ticks = element_line(colour = "black", size = 0.5),
        aspect.ratio = 2)
ggsave(filename = "Raw plots/Liver/ballo_lacto.pdf",
       plot = ballo_lacto,
       device = "pdf",
       height = 3,
       width = 5)
```

Fibrosis

```
fibr_pon1c <- DbNASH %>%   filter(complete.cases(Fibrosis)) %>%   ggpl
ot(aes(x = Fibrosis, y = PON1c, fill = Fibrosis)) +
  geom_boxplot(outlier.shape=NA, color = "black", size = 0.5) +
  stat_compare_means(label = "p", tip.length = 0, size = 4) +
  scale_fill_manual(values = c("#d42c7d", "#fbe1ae", "#84c922", "#eaea
ea", "#ff4f00")) +
  ylim(0, 300) +
  xlab("") +
  ylab("PON1 concentration (mg/L)") +
  theme_classic() +
  theme(axis.line = element_line(colour = "black", size = 0.5),
        panel.background = element_rect(fill = "white"),
        panel.grid.major = element_blank(),
        panel.grid.minor = element_blank(),
        axis.text = element_text(size = 12),
        axis.text.x = element_blank(),
        axis.ticks.x = element_blank(),
        axis.title = element_text(size = 14, face = "bold"),
        #legend.position =  "none",
        axis.ticks = element_line(colour = "black", size = 0.5),
        aspect.ratio = 2)
ggsave(filename = "Raw plots/Liver/fibr_pon1c.pdf",
       plot = fibr_pon1c,
       device = "pdf",
       height = 3,
       width = 5)
```

```
fibr_are <- DbNASH %>%   filter(complete.cases(Fibrosis)) %>%   ggplot
(aes(x = Fibrosis, y = ARE_act_2022, fill = Fibrosis)) +
  geom_boxplot(outlier.shape=NA, color = "black", size = 0.5) +
  stat_compare_means(label = "p", tip.length = 0, size = 4) +
  scale_fill_manual(values = c("#d42c7d", "#fbe1ae", "#84c922", "#eaea
ea", "#ff4f00")) +
  ylim(0, 300) +
  xlab("") +
  ylab("Arylesterease activity (U/L)") +
  theme_classic() +
  theme(axis.line = element_line(colour = "black", size = 0.5),
        panel.background = element_rect(fill = "white"),
        panel.grid.major = element_blank(),
        panel.grid.minor = element_blank(),
        axis.text = element_text(size = 12),
        axis.text.x = element_blank(),
        axis.ticks.x = element_blank(),
        axis.title = element_text(size = 14, face = "bold"),
        #legend.position =  "none",
        axis.ticks = element_line(colour = "black", size = 0.5),
        aspect.ratio = 2)
ggsave(filename = "Raw plots/Liver/fibr_are.pdf",
       plot = fibr_are,
       device = "pdf",
       height = 3,
       width = 5)

fibr_parax <- DbNASH %>%   filter(complete.cases(Fibrosis)) %>%   ggpl
ot(aes(x = Fibrosis, y = adj_Paraxonase, fill = Fibrosis)) +
  geom_boxplot(outlier.shape=NA, color = "black", size = 0.5) +
  #geom_point() +
  stat_compare_means(label = "p", tip.length = 0, size = 4) +
  scale_fill_manual(values = c("#d42c7d", "#fbe1ae", "#84c922", "#eaea
ea", "#ff4f00")) +
  ylim(0, 500) +
  xlab("") +
  ylab("Paraoxonasae activity (U/L)") +
  theme_classic() +
  theme(axis.line = element_line(colour = "black", size = 0.5),
        panel.background = element_rect(fill = "white"),
        panel.grid.major = element_blank(),
        panel.grid.minor = element_blank(),
        axis.text = element_text(size = 12),
        axis.text.x = element_blank(),
        axis.ticks.x = element_blank(),
        axis.title = element_text(size = 14, face = "bold"),
        #legend.position =  "none",
        axis.ticks = element_line(colour = "black", size = 0.5),
        aspect.ratio = 2)
ggsave(filename = "Raw plots/Liver/fibr_parax.pdf",
       plot = fibr_parax,
       device = "pdf",
       height = 3,
       width = 5)
```

```
fibr_lacto <- DbNASH %>%   filter(complete.cases(Fibrosis)) %>%   ggpl
ot(aes(x = Fibrosis, y = adj_Lactonase, fill = Fibrosis)) +
  geom_boxplot(outlier.shape=NA, color = "black", size = 0.5) +
  stat_compare_means(label = "p", tip.length = 0, size = 4) +
  scale_fill_manual(values = c("#d42c7d", "#fbe1ae", "#84c922", "#eaea
ea", "#ff4f00")) +
  ylim(0, 15) +
  xlab("") +
  ylab("Lactonase activity (U/L)") +
  theme_classic() +
  theme(axis.line = element_line(colour = "black", size = 0.5),
        panel.background = element_rect(fill = "white"),
        panel.grid.major = element_blank(),
        panel.grid.minor = element_blank(),
        axis.text = element_text(size = 12),
        axis.text.x = element_blank(),
        axis.ticks.x = element_blank(),
        axis.title = element_text(size = 14, face = "bold"),
        #legend.position =  "none",
        axis.ticks = element_line(colour = "black", size = 0.5),
        aspect.ratio = 2)
ggsave(filename = "Raw plots/Liver/fibr_lacto.pdf",
       plot = fibr_lacto,
       device = "pdf",
       height = 3,
       width = 5)
```

Biomarker

The final ROC plots were created with Metaboanalyst (www.metaboanalyst.ca).

```
# Create a binary outcome variable
Db$NASH_binary <- ifelse(Db$NAS_qual == 2, 1, 0)

pon1vars <- c("PON1c", "ARE_act_2022", "adj_Paraxonase", "adj_Lactonas
e")

roc_list <- lapply(pon1vars, function(var) {
  roc(Db$NASH_binary ~ Db[[var]], data = Db)
})

plot.roc(roc_list[[1]], main="ROC Curves for pon1vars")
sapply(2:length(pon1vars), function(i) {
  lines.roc(roc_list[[i]], col=i)
})
legend("bottomright", legend=pon1vars, col=1:length(pon1vars), lwd=2)
```

```
sapply(roc_list, auc)
```

PON1 polymorphism

Distribution

```
# Variable name change to avoid hyphen
names(Db)[names(Db) == "PON1-rs662-2022"] <- "PON1_polim"
Db <- Db %>%
  drop_na(`PON1_polim`)
```

All participants

```
group_totals <- Db %>%
  filter(!is.na(PON1_polim)) %>%
  dplyr::group_by(Group) %>%
  dplyr::summarise(total = n())

Db_percent <- Db %>%
  dplyr::group_by(PON1_polim, Group) %>%
  dplyr::summarise(count = n()) %>%
  dplyr::left_join(group_totals, by = "Group") %>%
  dplyr::mutate(percentage = (count / total) * 100)

gen_distr <- ggplot(data = Db_percent, aes(x = PON1_polim, y = percent
age, fill = Group)) +
  geom_col(position = "dodge", color = "black", alpha = 0.8) +
  labs(x = "PON1 polymorphism (rs662)", y = "Frequency (%)") +
  theme_minimal() +
  theme(
    plot.title = element_text(size = 16, face = "bold"),
    axis.text = element_text(size = 12),
    axis.title = element_text(size = 14, face = "bold"),
    panel.grid.major = element_blank(),
    panel.grid.minor = element_blank(),
    panel.background = element_blank(),
    axis.line = element_line(color = "black"),
    axis.ticks.x = element_blank(),
    aspect.ratio = 1.2/1
  )

ggsave(filename = "Raw plots/Genotypes/Distributiontotal.pdf",
       plot = gen_distr,
       device = "pdf",
       height = 3.2,
       width = 3.2)

gen_distr_g <- ggplot(data = Db, aes(x = PON1_polim, fill = Group)) +
  geom_bar(position = "fill", color = "black", alpha = 0.8) +
  labs(x = "PON1 polymorphism (rs662)", y = "Proportion of Participant
s") +
  theme_minimal() +
  theme(
    plot.title = element_text(size = 16, face = "bold"),
    axis.text = element_text(size = 12),
    axis.title = element_text(size = 14, face = "bold"),
    panel.grid.major = element_blank(),
    panel.grid.minor = element_blank(),
```

```
    panel.background = element_blank(),
    axis.line = element_line(color = "black"),
    axis.ticks.x = element_blank(),
    aspect.ratio = 1/0.7,
  )
ggsave(filename = "Raw plots/Genotypes/Frequency_gen_grouped.pdf",
       plot = gen_distr_g,
       device = "pdf",
       height = 3.2,
       width = 3.2)

contingency_table <- table(Db$PON1_polim, Db$Group)

chi2_test <- chisq.test(contingency_table)

print(chi2_test)

nas_totals <- Db %>%
  filter(!is.na(NAS_qual) & !is.na(PON1_polim)) %>%
  dplyr::group_by(NAS_qual) %>%
  dplyr::summarise(total = n())

Db_percent_NAS <- Db %>%
  filter(!is.na(NAS_qual) & !is.na(PON1_polim)) %>%
  dplyr::group_by(PON1_polim, NAS_qual) %>%
  dplyr::summarise(count = n()) %>%
  dplyr::left_join(nas_totals, by = "NAS_qual") %>%
  dplyr::mutate(percentage = (count / total) * 100) %>%
  dplyr::select(-total)  # to remove the total column

Db_percent_NAS$NAS_qual <- as.factor(Db_percent_NAS$NAS_qual)

gen_distr_NASH <- ggplot(data = Db_percent_NAS, aes(x = PON1_polim, y
= percentage, fill = NAS_qual)) +
  geom_col(position = "dodge", color = "black", alpha = 0.8) +
  labs(x = "PON1 polymorphism (rs662)", y = "Percentage (%)") +
  theme_minimal() +
  theme(
    plot.title = element_text(size = 16, face = "bold"),
    axis.text = element_text(size = 12),
    axis.title = element_text(size = 14, face = "bold"),
    panel.grid.major = element_blank(),
    panel.grid.minor = element_blank(),
    panel.background = element_blank(),
    axis.line = element_line(color = "black"),
    axis.ticks.x = element_blank(),
    aspect.ratio = 1.2/1
  )

ggsave(filename = "Raw plots/Genotypes/DistributionNASH.pdf",
       plot = gen_distr_NASH,
       device = "pdf",
       height = 3.2,
       width = 3.2)
```

```
contingency_table <- table(Db$PON1_polim, Db$NAS_qual)

chi2_test <- chisq.test(contingency_table)

print(chi2_test)
```

PON1 polymorphism, concentration and activity

```
stat.test <- Db %>%
  group_by(Group) %>%
  t_test(PON1c ~ PON1_polim) %>%
  adjust_pvalue(method = "bonferroni") %>%
  add_significance("p.adj")
stat.test
stat.test <- stat.test %>%
  add_xy_position(x = "Group", dodge = 0.8)
gen_pon1c <- ggplot(Db, aes(x = Group, y = PON1c, color = PON1_polim))
+
    geom_boxplot(outlier.shape=NA) +
    stat_pvalue_manual(stat.test,  label = "p", tip.length = 0) +
  theme(
    axis.line = element_line(color = "black"),
    panel.background = element_blank(),
    panel.grid = element_blank(),
    axis.text = element_text(size = 10, color = "black"),
    legend.key = element_blank(),
    aspect.ratio = 1.3/1) +
  ylim(0,300)
ggsave(filename = "Raw plots/Genotypes/Gen_pon1c.pdf",
       plot = gen_pon1c,
       device = "pdf",
       height = 3.5,
       width = 3.5)

stat.test <- Db %>%
  group_by(Group) %>%
  t_test(ARE_act_2022 ~ PON1_polim) %>%
  adjust_pvalue(method = "bonferroni") %>%
  add_significance("p.adj")
stat.test
stat.test <- stat.test %>%
  add_xy_position(x = "Group", dodge = 0.8)
gen_are <- ggplot(Db, aes(x = Group, y = ARE_act_2022, color = PON1_po
lim)) +
    geom_boxplot(outlier.shape=NA) +
  geom_point(alpha=0.5) +
    stat_pvalue_manual(stat.test,  label = "p", tip.length = 0) +
  theme(
    axis.line = element_line(color = "black"),
    panel.background = element_blank(),
    panel.grid = element_blank(),
    axis.text = element_text(size = 10, color = "black"),
    legend.key = element_blank(),
    aspect.ratio = 1.3/1)
```

```
ggsave(filename = "Raw plots/Genotypes/Gen_are.pdf",
       plot = gen_are,
       device = "pdf",
       height = 3.5,
       width = 3.5)

stat.test <- Db %>%
  group_by(Group) %>%
  t_test(adj_Paraxonase ~ PON1_polim) %>%
  adjust_pvalue(method = "bonferroni") %>%
  add_significance("p.adj")
stat.test
stat.test <- stat.test %>%
  add_xy_position(x = "Group", dodge = 0.8)
gen_parax <- ggplot(Db, aes(x = Group, y = adj_Paraxonase, color = PON
1_polim)) +
    geom_boxplot(outlier.shape=NA) +
    stat_pvalue_manual(stat.test,  label = "p", tip.length = 0) +
  theme(
    axis.line = element_line(color = "black"),
    panel.background = element_blank(),
    panel.grid = element_blank(),
    axis.text = element_text(size = 10, color = "black"),
    legend.key = element_blank(),
    aspect.ratio = 1.3/1)
ggsave(filename = "Raw plots/Genotypes/Gen_parax.pdf",
       plot = gen_parax,
       device = "pdf",
       height = 3.5,
       width = 3.5)

stat.test <- Db %>%
  group_by(Group) %>%
  t_test(adj_Lactonase ~ PON1_polim) %>%
  adjust_pvalue(method = "bonferroni") %>%
  add_significance("p.adj")
stat.test
stat.test <- stat.test %>%
  add_xy_position(x = "Group", dodge = 0.8)
gen_lacton <- ggplot(Db, aes(x = Group, y = adj_Lactonase, color = PON
1_polim)) +
    geom_boxplot(outlier.shape=NA) +
    stat_pvalue_manual(stat.test,  label = "p", tip.length = 0) +
  theme(
    axis.line = element_line(color = "black"),
    panel.background = element_blank(),
    panel.grid = element_blank(),
    axis.text = element_text(size = 10, color = "black"),
    legend.key = element_blank(),
    aspect.ratio = 1.3/1)
ggsave(filename = "Raw plots/Genotypes/Gen_lacton.pdf",
       plot = gen_lacton,
       device = "pdf",
```

```
       height = 3.5,
       width = 3.5)
```

Table S2. P-values from Shapiro-Wilk normality test for PON1-related variables.

	p-value
PON1 concentration	< 0.001
Arylesterase activity	< 0.001
Paraoxonase activity	< 0.001
Lactonase activity	0.656

Table S3. Age and sex were tested as confounding factors for PON1-related variables.

	Age		Sex	
	**p-value**	**Rho**	**p-value**	**Statistic**
PON1 concentration	0.818	0.07	0.215	119640.5
Arylesterase activity	0.790	-0.01	0.130	151785.0
Paraoxonase activity	0.115	-0.02	0.011	188477.0
Lactonase activity	0.003	-0.09	0.224	119402.0

Spearman's rho measures the strength and direction of the association between the two continuous variables. Statistic is the W statistic from the Wilcoxon test, which is a rank sum.

Table S4. Linear regression analysis showing the relationship between PON1 variables and metabolic syndrome, type 2 diabetes mellitus, hypertension, and dyslipidemia as a set.

	Estimate	Std error	p-value	r-squared
PON1 concentration	0.03	0.11	0.80	0.01
Arylesterase activity	0.01	0.02	0.80	0.12
Paraoxonase activity	0.01	0.04	0.74	0.14
Lactonase activity	0.00	0.00	0.80	0.17

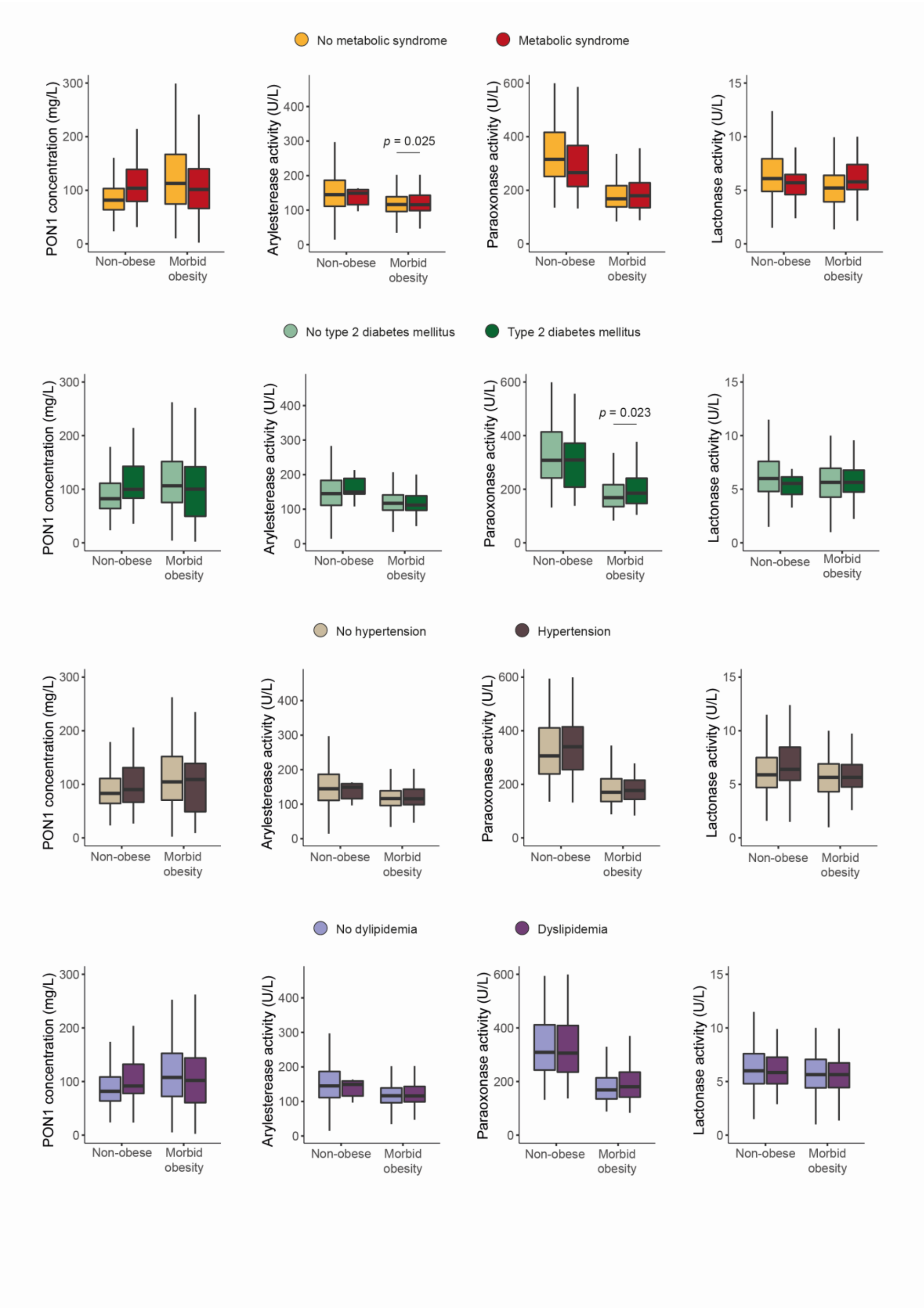

Figure S1. Paraoxonase-1 (PON1)-related variables in non-obese individuals and morbidly obese patients segregated according to comorbidities.

Table S5. Clinical, analytical, and histological characteristics of morbidly obese patients and different degrees of liver severity.

	**Non-NASH (n=347)**	**Uncertain NASH (n=311)**	**NASH (n=173)**	**p-value**
Female sex, n (%)	258 (74.4)	217 (70.0)	131 (75.7)	0.303
Age (years)	48 (40 - 55)	50 (42 - 56)	49 (41 - 57)	0.121
BMI (kg/m^2)	44.5 (40.9 - 48.9)	44.6 (40.9 - 48.8)	43.3 (40.3 - 48.6)	0.479
Waist circumference (cm)	132 (122 - 142)	130 (121 - 140)	130 (121 - 140)	0.731
T2DM (n (%))	85 (24.5)	81 (26.0)	44 (25.4)	0.900
HT (n (%))	144 (41.5)	133 (42.8)	73 (42.2)	0.947
DLP (n (%))	70 (20.2)	82 (26.4)	44 (25.4)	0.142
Metabolic syndrome (n (%))	145 (41.8)	122 (39.2)	70 (40.5)	0.800
Glucose (mmol/L)	6.6 (5.5 - 8.3)	6.9 (5.7 - 8.5)	6.8 (5.5 - 8.2)	0.288
Insulin (pmol/L)	67.2 (36.2 - 125)	75.5 (46.1 - 124.9)	61.1 (37.5 - 104.2)	0.068
HOMA-IR	3.1 (1.5 - 6.3)	3.5 (2 - 6)	3 (1.7 - 4.9)	0.119
Triglycerides (mmol/L)	1.4 (1.1 - 2)	1.5 (1.2 - 2)	1.6 (1.2 - 2)	0.676
Total cholesterol (mmol/L)	4.1 (3.5 - 4.7)	4.1 (3.5 - 4.8)	4 (3.6 - 4.7)	0.999
HDL cholesterol (mmol/L)	1 (0.8 - 1.2)	1 (0.8 - 1.2)	0.9 (0.8 - 1.1)	0.060
LDL cholesterol (mmol/L)	2.5 (2 - 3.1)	2.4 (1.9 - 3)	2.5 (1.9 - 3.1)	0.356
VLDL cholesterol (mmol/L)	0.7 (0.5 - 0.9)	0.7 (0.5 - 0.9)	0.7 (0.5 - 0.9)	0.676
ALT (µKat/L)	0.6 (0.4 - 0.9)	0.6 (0.4 - 0.9)	0.6 (0.4 - 0.8)	0.993
AST (µKat/L)	0.6 (0.4 - 0.8)	0.6 (0.4 - 0.8)	0.6 (0.4 - 0.8)	0.662
GGT (µKat/L)	0.4 (0.3 - 0.6)	0.4 (0.2 - 0.6)	0.4 (0.3 - 0.5)	0.596
Steatosis grade (%)	5 (1 - 10)	15 (10 - 32.50)	50 (40 - 70)	<0.001
Steatosis score, n (%))				<0.001
0	249 (72)	63 (20)	-	
1	91 (26)	170 (55)	19 (11)	
2	7 (2)	74 (24)	92 (53)	
3	-	4 (1)	62 (36)	
Lobular inflammation, n (%)				< 0.001

0	99 (29)	15 (5)	-	
1	209 (61)	167 (54)	52 (30)	
2	34 (9.9)	103 (33.3)	96 (55.5)	
3	-	24 (7.8)	25 (14.5)	
Ballooning, n (%)				< 0.001
0	260 (76)	90 (29)	6 (4)	
1	76 (22.3)	154 (49.7)	83 (48.0)	
2	5 (1.5)	66 (21.3)	84 (48.6)	
Fibrosis, n (%)				< 0.001
0	70 (21)	17 (6)	12 (7)	
1	136 (40)	107 (35)	36 (21)	
2	112 (33)	137 (45)	66 (39)	
3	22 (7)	42 (14)	52 (31)	
4	-	1 (0)	3 (2)	
NAFLD Activity Score, n (%)				< 0.001
0	49 (14)	-	-	
1	128 (37)	-	-	
2	170 (49)	-	-	
3	-	183 (59)	-	
4	-	128 (41)	-	
5	-	-	100 (58)	
6	-	-	54 (31)	
7	-	-	17 (10)	
8	-	-	2 (1)	

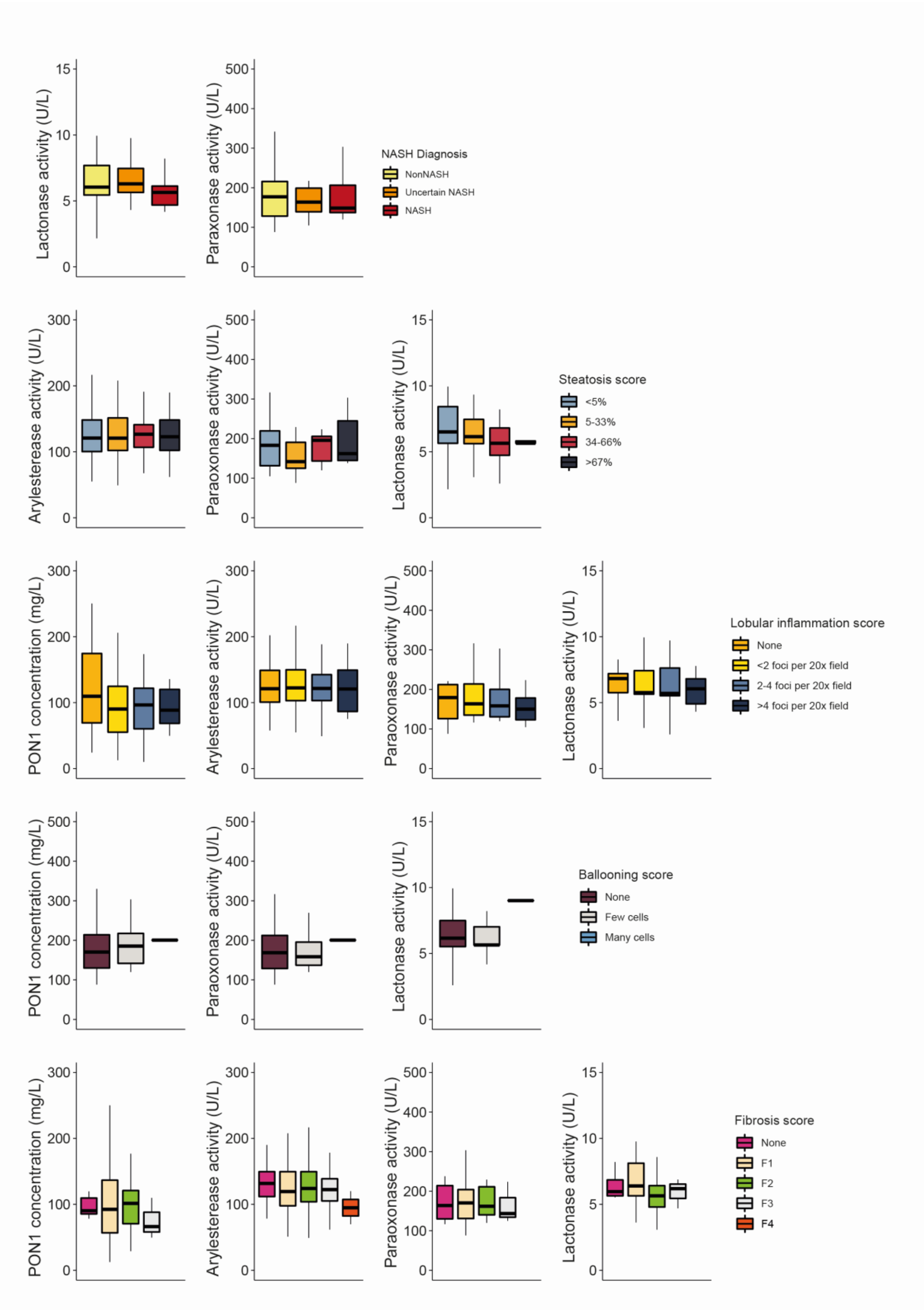

Figure S2. Paraoxonase-1 (PON1)-related variables in morbidly obese patients segregated according to the histopathological characteristics of liver biopsies.

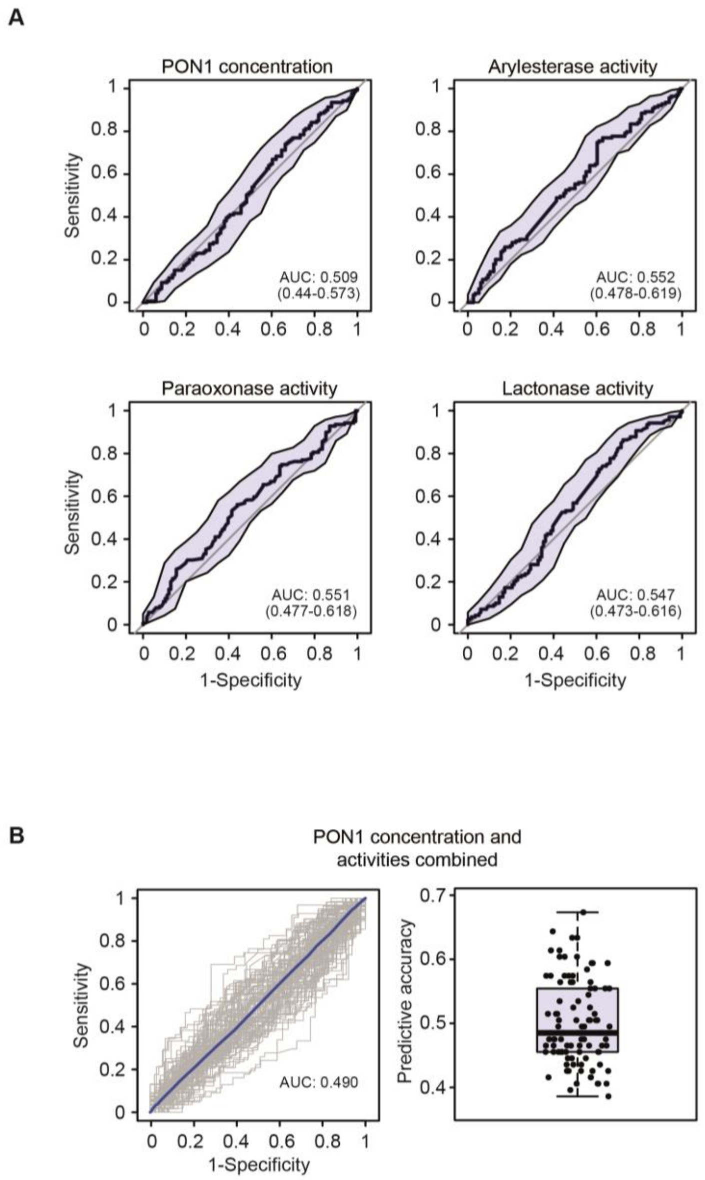

Figure S3. Paraoxonase-1 (PON1) concentration and activities were not able to predict NASH in morbidly obese patients. (A) Receiver operating characteristics (ROC) of PON1-related variables. (B) ROC from the predictive model built with linear supervised vector machine (SVM) and its accuracy.
